# Single-point insulin sensitivity estimator and atrial fibrillation: association and incremental discriminative value in a hospital-based population

**DOI:** 10.3389/fendo.2026.1862319

**Published:** 2026-07-01

**Authors:** Yuzhou Liu, Zhe Meng, Xijia Wang, Hongrui Chen

**Affiliations:** Department of Cardiology, The First Affiliated Hospital of Zhengzhou University, Zhengzhou, Henan, China

**Keywords:** AF identification, atrial fibrillation, discriminative value, hospital-based population, insulin resistance surrogate, SPISE

## Abstract

**Background:**

The single-point insulin sensitivity estimator (SPISE), derived from triglycerides, high-density lipoprotein cholesterol, and body mass index, has emerged as an easily calculated marker of insulin sensitivity. Its association with atrial fibrillation (AF) has not been well defined.

**Methods:**

This retrospective cross-sectional study included 1,122 hospitalized participants, including 309 without AF, 409 with paroxysmal AF, and 404 with persistent AF. The association between SPISE and AF was examined with logistic regression by modeling SPISE continuously, per standard deviation (SD), and by quartiles. Restricted cubic splines were used to assess the shape of the association. Candidate variables were reduced by LASSO and then entered into univariable and multivariable logistic regression to construct the AF classification model. Model performance was evaluated with discrimination, calibration, decision-curve analysis, repeated 10-fold cross-validation, and bootstrap optimism correction. The incremental discriminative value of SPISE was assessed by changes in area under the receiver operating characteristic curve (AUC), net reclassification improvement (NRI), and integrated discrimination improvement (IDI).

**Results:**

Lower SPISE was associated with AF after multivariable adjustment. In the fully adjusted model, the corresponding odds ratios were 0.68 (0.61–0.76) per unit increase and 0.56 (0.47–0.66) per SD increase. Compared with the lowest quartile, the highest quartile showed lower odds of AF, with an odds ratio of 0.36 (0.23–0.58). The relationship was predominantly linear. The AF classification model achieved an AUC of 0.859 (0.836–0.882), and the mean cross-validated C-statistic was 0.855. Adding SPISE increased AUC from 0.843 to 0.862 and significantly improved both NRI and IDI; however, the absolute AUC gain was small, although NRI and IDI improved statistically.

**Conclusions:**

Lower SPISE was associated with AF after multivariable adjustment and added discriminative information beyond conventional clinical variables in this hospital-based sample. Given the small absolute improvement in AUC, SPISE should be interpreted as an adjunctive marker of AF-related metabolic phenotype rather than a stand-alone component of routine AF assessment. Prospective studies are needed to determine whether SPISE is related to incident AF.

## 1 Introduction

Among sustained cardiac arrhythmias, atrial fibrillation (AF) is the most frequently encountered and its burden continues to expand worldwide (1). Population aging, together with the increasing prevalence of obesity and metabolic disorders, has contributed to the rising number of individuals affected by AF, making it an important public health challenge (2, 3). AF is closely linked to stroke, heart failure, and all-cause mortality (4), and it also imposes substantial demands on healthcare resources (5). Against this background, identifying modifiable factors related to AF remains important for improving the recognition of AF-related phenotypes and guiding further clinical evaluation (6).

Growing evidence has implicated metabolic disturbance in both the initiation and maintenance of AF. Obesity, type 2 diabetes, and related metabolic abnormalities are all associated with AF (7), and impaired insulin sensitivity has been regarded as one of their shared pathophysiological features (8). Experimental work suggests that reduced insulin sensitivity may be associated with AF-related atrial changes through lipid accumulation, inflammation, fibrosis, and both electrical and structural remodeling of the atrium (9, 10). Observational studies have likewise linked insulin resistance to AF independently of several conventional cardiovascular risk factors (11). Even so, the available evidence remains inconclusive. Recent Mendelian randomization analyses have not consistently supported a clear causal relationship between genetically predicted insulin resistance and AF (12). One possible reason for this inconsistency is the marked variation in how insulin sensitivity has been assessed across studies (13).

The hyperinsulinemic-euglycemic clamp is generally regarded as the reference approach for evaluating insulin sensitivity, yet its invasive nature, technical complexity, and cost limit its use in routine practice and large-scale studies (14, 15). HOMA-IR is more widely applied, but it still depends on insulin measurement and may be less suitable in individuals with impaired pancreatic function (16). The single-point insulin sensitivity estimator (SPISE) has recently emerged as a simple surrogate index derived from triglycerides (TG), high-density lipoprotein cholesterol (HDL-C), and body mass index (BMI) (17, 18). Because it does not require insulin testing, SPISE may be better suited to clinical and population-based research settings. Although SPISE has attracted attention in studies of dysglycemia and metabolic syndrome (19, 20), direct evidence linking SPISE to AF remains limited.

Accordingly, the present study examined the association between SPISE and AF in a hospital-based population. We further evaluated the shape of this association and the incremental discriminative value of SPISE for identifying AF beyond clinical reference models.

## 2 Materials and methods

### 2.1 Data source and ethical approval

This study was a secondary analysis of publicly released anonymized patient-level dataset from the study by Li et al. (21). The original study included hospitalized patients from the First Hospital of Jilin University between January 1, 2023 and February 29, 2024, and the dataset was released as Supporting Information with the corresponding PLOS ONE publication. The original article stated that all relevant data were available within the manuscript and its Supporting Information files. The original article was distributed under the terms of the Creative Commons Attribution License, which permits unrestricted use, distribution, and reproduction in any medium, provided that the original author and source are credited.

The present analysis was based only on this publicly released anonymized Supporting Information dataset. It did not involve privately obtained data, institutional data transfer, or additional access to medical records from the source hospital. No restricted dataset, privately transferred patient-level data, or identifiable clinical records were received or requested from the original investigators or the source institution.

According to the original publication, the study was approved by the Ethics Committee of the First Hospital of Jilin University (No. 2024−527) (21), and informed consent was waived because of the retrospective design. The present work involved no patient contact, no clinical intervention, no access to identifiable clinical records, no private data transfer, and no new patient-level data collection. The original study was cited as the data source, and Li et al. (21) were acknowledged for making the anonymized dataset available as Supporting Information.

### 2.2 Study population

**Figure 1:**
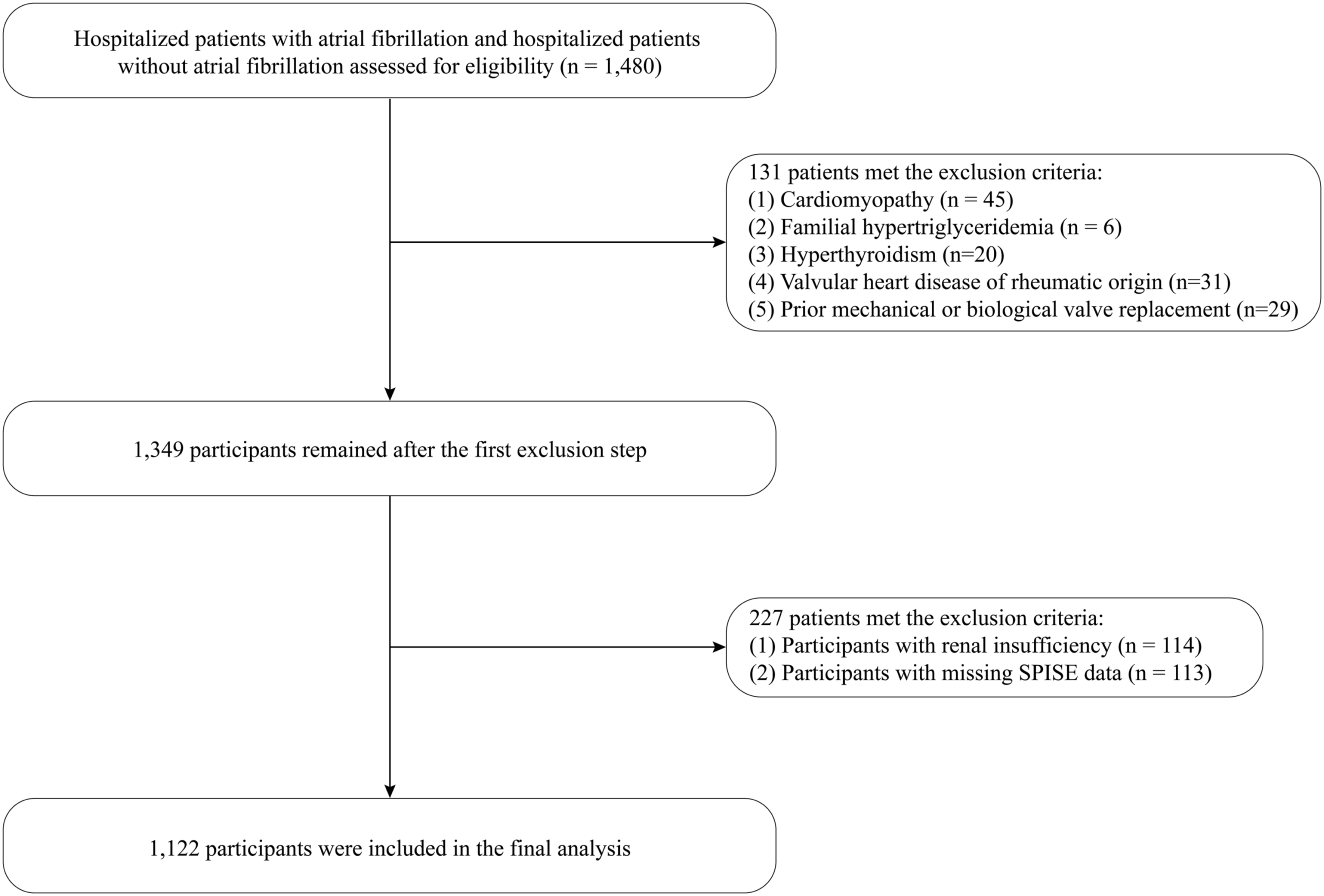
Flowchart of participant selection in the present study.

The study cohort was derived in two screening steps (**Figure 1**). Initially, 1,480 hospitalized patients, including AF cases and non-AF inpatients, were reviewed. At the first step, 131 individuals were removed because of cardiomyopathy, familial hypertriglyceridemia, hyperthyroidism, rheumatic valvular heart disease, or previous mechanical or biological valve replacement, leaving 1,349 participants. A second screening step excluded 114 participants with renal insufficiency and 113 with missing SPISE data. The final study population therefore consisted of 1,122 participants, including 813 participants with AF and 309 without AF. The non-AF group consisted of hospitalized participants without documented AF, rather than community-based healthy controls. Primary hospitalization diagnoses for the non-AF participants were not provided in the released dataset. No further exclusion criteria based on lipid- or liver-metabolism–altering conditions could be applied because such diagnoses or exposures were not separately recorded in the released dataset.

### 2.3 Assessment of SPISE and other study variables

Study variables were retrieved from the source cohort database and covered clinical characteristics, laboratory measurements, and echocardiographic parameters. Clinical characteristics included age, sex, height, weight, smoking, drinking, hypertension, DM, CHD, and stroke. Height and weight were extracted as recorded anthropometric variables from the source cohort database and were used to calculate BMI. No additional anthropometric measurements were performed for the present secondary analysis. Laboratory data included RBC, PLT, ALT, AST, TC, TG, HDL-C, LDL-C, SCr, UA, and FBG. Echocardiographic parameters included LVEF, LVEDD, LAD, and LVDD. Information on obstructive sleep apnea, physical activity, liver cirrhosis, active malignancy, pregnancy, dietary supplement use, and medication use, including lipid-lowering therapy, specific antihypertensive agents, and glucose-lowering treatment, was not available in the released dataset.

AF subtypes were classified according to the criteria adopted in the source cohort. Paroxysmal AF was defined as AF episodes lasting no more than 7 days, whereas persistent AF referred to episodes lasting longer than 7 days (1, 21). For the subsequent analyses, paroxysmal AF and persistent AF were combined into the AF group, and participants without AF were treated as the reference group.

SPISE was calculated as 600 × HDL-C^0.185/(TG^0.2 × BMI^1.338), with HDL-C and TG expressed in mg/dL and BMI in kg/m² (17, 18). For comparison with other non-insulin-based insulin resistance surrogate markers, the TyG index and TG/HDL-C ratio were additionally calculated. The TyG index was calculated as ln[TG (mg/dL) × FBG (mg/dL)/2]. The TG/HDL-C ratio was calculated as TG divided by HDL-C using the same lipid concentration units.

### 2.4 Statistical analysis

The analyses proceeded in three steps. Baseline characteristics were first compared across the non-AF, paroxysmal AF, and persistent AF groups; continuous variables were summarized according to their distributions and compared with parametric or rank-based tests as appropriate, whereas categorical variables were compared with χ² tests. All subsequent association and classification analyses treated AF status as a binary outcome.

SPISE was examined in relation to AF in logistic regression models, with SPISE entered per unit, per SD, and by quartiles. Restricted cubic splines with four knots were fitted in the logistic regression framework to describe the shape of the association between SPISE and AF and to assess potential nonlinearity. Odds ratios in the spline analysis were presented using SPISE = 5.803 as the reference value.

Because SPISE differed across AF subtypes in the baseline comparison, exploratory subtype-specific analyses were additionally performed. Paroxysmal AF and persistent AF were first examined separately using non-AF participants as the reference group. An AF-only comparison was then performed between paroxysmal and persistent AF. These analyses used logistic regression and were adjusted for the available covariates used in the main multivariable models.

To address potential interpretive overlap between SPISE and its component variables, we performed component-sensitivity analyses. Spearman correlations were calculated between SPISE and BMI, TG, HDL-C, FBG, and DM status. Collinearity was assessed using variance inflation factors (VIFs). We then compared the broad covariate reference model with three expanded models: the basic covariate model plus SPISE, the basic covariate model plus the individual SPISE components (BMI, TG, and HDL-C), and the basic covariate model plus both SPISE and its components. The basic covariate model included sex, smoking, drinking, DM, hypertension, CHD, LAD, LVEF, LVEDD, LVDD, AST, ALT, SCr, PLT, and RBC. RBC may reflect oxygen-carrying capacity and anemia-related clinical status, whereas PLT may reflect platelet-related and thrombo-inflammatory status. Complete blood count parameters, including red blood cell- and platelet-related indices, have been evaluated in previous AF-related studies and reviews. Therefore, RBC and PLT were retained as adjustment covariates in Model 3.

For construction of the AF classification model, candidate variables for LASSO regression were selected from clinically relevant variables available in the released dataset, including age, sex, smoking, drinking, hypertension, DM, CHD, stroke, LAD, LVEF, LVEDD, LVDD, SCr, ALT, AST, RBC, PLT, and SPISE. Continuous variables were standardized before penalized regression, and binary variables were coded as 0/1. LASSO logistic regression was performed using 10-fold cross-validation. Because the LASSO step was used as a preliminary variable-reduction procedure rather than as the final model, we selected the penalty value that retained the largest set of non-zero candidate variables from the cross-validated LASSO path. Variables retained by LASSO were then evaluated using univariable logistic regression, followed by multivariable logistic regression to construct the final AF classification model. The univariable step was used as an intermediate assessment of the LASSO-retained variables before multivariable modeling.

Model performance was evaluated by discrimination, calibration, Brier score, and decision-curve analysis. Discrimination was assessed using the area under the receiver operating characteristic curve (AUC). Calibration was described using the calibration intercept, calibration slope, and calibration curve. A nomogram was generated as an exploratory graphical representation of the fitted AF classification model rather than as a validated clinical prediction instrument. Internal validation was performed using repeated 10-fold cross-validation for the specified final AF classification model. Bootstrap optimism correction with 1000 resamples was additionally performed. In each bootstrap sample, the specified final model was refitted, and optimism was estimated by comparing model performance in the bootstrap sample with performance when the bootstrap-fitted model was applied to the original dataset. These internal validation analyses were conducted after model specification and did not repeat the full variable-selection process within each resampling iteration.

The incremental discriminative value of SPISE was assessed by changes in AUC, category-free net reclassification improvement (NRI), and integrated discrimination improvement (IDI). Category-free NRI was used because no established risk categories are available for identifying AF in this hospital-based cross-sectional setting; therefore, no risk thresholds were applied. For the incremental discriminative analysis, two reference models were used. The broad covariate reference model was constructed from the available non-SPISE covariates used in the fully adjusted association model, including lifestyle, comorbidity, echocardiographic, hepatic, renal, and hematologic variables. BMI, TG, and HDL-C were not included because they are mathematical components of SPISE. The LASSO-derived clinical reference model consisted of the non-SPISE predictors retained in the final multivariable AF classification model, including SCr, ALT, AST, LAD, LVDD, and DM. SPISE was then added to each reference model to assess its incremental discriminative value. To compare SPISE with other insulin-resistance surrogate markers, single-marker ROC analyses were performed for SPISE, TyG index, and TG/HDL-C ratio. Model-based comparisons were then performed by adding each marker separately to the broad covariate reference model and comparing the resulting AUCs with that of the reference model.

Several sensitivity analyses were performed to examine the robustness of the association between SPISE and AF, particularly in relation to lipid-related, liver-related, and medication-related confounding. Because medication use, including lipid-lowering therapy, was not available in the released dataset, additional analyses were performed using available clinical and laboratory variables. These analyses included repeating the fully adjusted model after removing RBC and PLT, applying additional adjustment for TC, LDL-C, and FBG, excluding participants with CHD, stroke, or DM, and excluding participants with extreme TG, HDL-C, ALT, or AST values. Extreme TG values were defined as values above the 99th percentile, extreme HDL-C values as values below the 1st percentile or above the 99th percentile, and extreme liver-enzyme values as ALT or AST values above the 99th percentile.

Additional robustness analyses were then performed to address available markers of clinically relevant liver-enzyme abnormality, altered lipid status, extreme body size, and cardiometabolic comorbidity burden. These analyses excluded participants with ALT or AST >80 U/L, participants with ALT or AST >80 U/L or extreme TG/HDL-C values, participants with LDL-C <1.8 mmol/L, participants with extreme BMI values defined as values below the 1st percentile or above the 99th percentile, and participants with CHD, stroke, DM, or hypertension.

E-values were also calculated to quantify the minimum strength of association that an unmeasured confounder would need to have with both SPISE and AF, conditional on the measured covariates, to explain away the observed association. Because AF was common in this hospital-based analytic sample, E-values were calculated after approximate conversion of odds ratios to the risk-ratio scale.

All analyses were conducted using R software, version 4.4.2 (R Foundation for Statistical Computing, Vienna, Austria). A two-sided P value < 0.05 was considered statistically significant.

## 3 Results

### 3.1 Baseline characteristics across AF groups

The analysis included 1,122 participants, of whom 309 were classified as non-AF, 409 as paroxysmal AF, and 404 as persistent AF (**Table 1**). Age did not vary across groups (P = 0.930). Drinking, TC, and LDL-C were also similar among the three groups (all P > 0.05).

**Table 1:** Baseline characteristics of the study population according to atrial fibrillation status.

Variables	Total (n = 1122)	Non-AF (n = 309)	Paroxysmal AF (n = 409)	Persistent AF (n = 404)	P value
Age, years	61.00 (55.00, 68.00)	61.00 (55.00, 69.00)	61.00 (56.00, 68.00)	62.00 (55.75, 68.00)	0.930
Male, n (%)	634 (56.51)	137 (44.34)	224 (54.77)	273 (67.57)	< 0.001
Smoking, n (%)	92 (8.20)	34 (11.00)	23 (5.62)	35 (8.66)	0.031
Drinking, n (%)	93 (8.29)	24 (7.77)	26 (6.36)	43 (10.64)	0.079
Hypertension, n (%)	545 (48.57)	116 (37.54)	226 (55.26)	203 (50.25)	< 0.001
DM, n (%)	213 (18.98)	35 (11.33)	103 (25.18)	75 (18.56)	< 0.001
CHD, n (%)	232 (20.68)	54 (17.48)	106 (25.92)	72 (17.82)	0.005
Stroke, n (%)	113 (10.07)	22 (7.12)	32 (7.82)	59 (14.60)	< 0.001
BMI, kg/m²	25.64 (23.38, 27.80)	24.44 (22.10, 27.01)	25.88 (23.69, 27.92)	26.09 (23.67, 28.21)	< 0.001
TC, mmol/L	4.40 (3.75, 5.13)	4.45 (3.78, 5.18)	4.40 (3.78, 5.13)	4.36 (3.68, 5.04)	0.406
TG, mmol/L	1.42 (1.07, 1.98)	1.11 (0.83, 1.64)	1.68 (1.34, 2.29)	1.36 (1.04, 1.92)	< 0.001
HDL-C, mmol/L	1.05 (0.90, 1.21)	1.11 (0.96, 1.30)	1.02 (0.88, 1.14)	1.04 (0.90, 1.21)	< 0.001
LDL-C, mmol/L	2.75 (2.28, 3.31)	2.84 (2.28, 3.36)	2.73 (2.30, 3.29)	2.71 (2.23, 3.29)	0.201
ALT, U/L	19.90 (14.60, 28.78)	16.90 (12.70, 24.30)	20.30 (15.20, 30.60)	21.35 (15.60, 30.22)	< 0.001
AST, U/L	20.80 (17.00, 25.60)	19.80 (16.30, 23.50)	20.50 (16.70, 25.50)	21.90 (18.30, 28.45)	< 0.001
RBC, ×10¹²/L	4.84 ± 1.22	4.72 ± 2.13	4.81 ± 0.54	4.95 ± 0.57	0.042
PLT, ×10^9^/L	229.77 ± 60.20	236.89 ± 59.97	230.73 ± 60.66	223.35 ± 59.36	0.011
SCr, μmol/L	69.30 (59.52, 80.07)	64.00 (56.60, 74.40)	68.50 (59.20, 80.00)	73.05 (62.48, 82.70)	< 0.001
Uric acid, μmol/L	350 (295, 415.75)	320 (273, 366)	351(293, 420)	377.50 (310, 446.25)	< 0.001
FBG, mmol/L	5.38 (4.91, 6.12)	5.20 (4.79, 5.60)	5.58 (5.06, 6.57)	5.39 (4.92, 6.17)	< 0.001
SPISE	6.09 ± 1.53	6.84 ± 1.72	5.63 ± 1.18	5.98 ± 1.47	< 0.001
LAD, mm	38.00 (33.00, 42.00)	32.00 (30.00, 36.00)	36.00 (33.00, 40.00)	42.00 (40.00, 46.00)	< 0.001
LVEF, %	62.00 (58.00, 64.00)	63.00 (62.00, 65.00)	63.00 (60.00, 64.00)	59.00 (54.00, 63.00)	< 0.001
LVDD, n (%)	602 (53.70)	64 (20.78)	171 (41.81)	367 (90.84)	< 0.001
LVEDD, mm	48.00 (45.00, 51.00)	46.00 (43.00, 49.00)	48.00 (45.00, 50.00)	49.50 (46.00, 54.00)	< 0.001

P values represent overall comparisons among the non-AF, paroxysmal AF, and persistent AF groups. Continuous variables were compared using parametric or rank-based tests as appropriate, and categorical variables were compared using χ² tests. AF, atrial fibrillation; BMI, body mass index; ALT, alanine aminotransferase; AST, aspartate aminotransferase; CHD, coronary heart disease; DM, diabetes mellitus; FBG, fasting blood glucose; HDL-C, high-density lipoprotein cholesterol; LAD, left atrial diameter; LDL-C, low-density lipoprotein cholesterol; LVDD, left ventricular diastolic dysfunction; LVEDD, left ventricular end-diastolic diameter; LVEF, left ventricular ejection fraction; PLT, platelet count; RBC, red blood cell count; SCr, serum creatinine; TC, total cholesterol; TG, triglyceride; SPISE, single-point insulin sensitivity estimator.

In contrast, several other variables were unevenly distributed across AF categories. The AF groups included a greater proportion of men and showed higher frequencies of hypertension, DM, CHD, and prior stroke than the non-AF group. Higher TG, ALT, AST, SCr, UA, and FBG, together with lower HDL-C, were also observed in participants with AF. Routine hematologic parameters also differed across groups, with higher RBC and lower PLT levels observed across AF categories compared with the non-AF group. Echocardiographic measures showed larger LAD and LVEDD, a higher prevalence of LVDD, and lower LVEF in the AF groups. SPISE differed across the three groups as well, being highest in the non-AF group and lowest in the paroxysmal AF group (P < 0.001).

### 3.2 Association of SPISE with AF

Logistic regression analyses identified an inverse relationship between SPISE and AF (**Table 2**). When SPISE was analyzed as a continuous variable, the association remained materially unchanged across the crude, partially adjusted, and fully adjusted models. Under full adjustment, the odds ratio for AF was 0.68 (0.61–0.76; P < 0.001) for each 1-unit higher SPISE. The same inverse pattern was evident when SPISE was examined on an SD scale. In the fully adjusted model, a 1-SD increment in SPISE corresponded to an odds ratio of 0.56 (0.47–0.66; P < 0.001).

**Table 2:** Associations of SPISE with atrial fibrillation in logistic regression models.

Characteristic	Model1		Model2		Model3	
	OR(95%CI)	P-value	OR(95%CI)	P-value	OR(95%CI)	P-value
Continuous SPISE (per unit)	0.64 (0.58~0.70)	<0.001	0.69 (0.62~0.77)	<0.001	0.68 (0.61~0.76)	<0.001
Continuous SPISE (per SD)	0.51 (0.44~0.58)	<0.001	0.57 (0.48~0.67)	<0.001	0.56 (0.47~0.66)	<0.001
Q1	Ref.		Ref.		Ref.	
Q2	1.16 (0.75~1.79)	0.517	1.39 (0.85~2.28)	0.193	1.42 (0.86~2.36)	0.175
Q3	0.61 (0.41~0.91)	0.015	0.76 (0.48~1.21)	0.243	0.75 (0.46~1.21)	0.232
Q4	0.25 (0.17~0.36)	<0.001	0.36 (0.23~0.55)	<0.001	0.36 (0.23~0.58)	<0.001
P for trend	0.59 (0.52~0.67)	<0.001	0.67 (0.58~0.77)	<0.001	0.68 (0.58~0.79)	<0.001

Model 1: Unadjusted.

Model 2: Adjusted for age, sex, and LAD.

Model 3: Adjusted for age, sex, smoking, hypertension, drinking, DM, CHD, LAD, LVEF, LVEDD, LVDD, Scr, ALT, AST, RBC and PLT. AF, atrial fibrillation; ALT, alanine aminotransferase; AST, aspartate aminotransferase; CHD, coronary heart disease; DM, diabetes mellitus; LAD, left atrial diameter; LVDD, left ventricular diastolic dysfunction; LVEDD, left ventricular end-diastolic diameter; LVEF, left ventricular ejection fraction; SCr, Serum creatinine; RBC, red blood cell; PLT, platelet; SPISE, single point insulin sensitivity estimator; CI, confidence interval; OR, odds ratio.

Results from the quartile analysis showed the clearest separation in the highest SPISE quartile. Relative to Q1, Q4 was associated with lower odds of AF after full adjustment, with an odds ratio of 0.36 (0.23–0.58; P < 0.001), whereas Q2 and Q3 were not statistically significant. However, the continuous model, per-SD model, and trend test all supported an overall inverse association between SPISE and AF (P for trend < 0.001).

### 3.3 Exploratory subtype-specific analysis

To further examine the lower SPISE level observed in the paroxysmal AF group at baseline, exploratory subtype-specific analyses were performed (**Table 3**). Compared with the non-AF group, higher SPISE was associated with lower odds of both paroxysmal AF and persistent AF. The adjusted ORs per 1-unit higher SPISE were 0.61 (0.53–0.69) for paroxysmal AF and 0.71 (0.60–0.84) for persistent AF. The corresponding ORs per 1-SD higher SPISE were 0.47 (0.38–0.57) and 0.59 (0.45–0.77), respectively. Among participants with AF, higher SPISE was associated with lower odds of paroxysmal AF relative to persistent AF, with adjusted ORs of 0.62 (0.53–0.73) per 1-unit increase and 0.48 (0.38–0.62) per 1-SD increase.

**Table 3:** Exploratory subtype-specific associations between SPISE and atrial fibrillation subtype.

Comparison	SPISE scale	N/events	Adjusted OR (95% CI)	Adjusted P
Paroxysmal AF vs. non-AF	per unit	718/409	0.61 (0.53–0.69)	<0.001
	per SD	718/409	0.47 (0.38–0.57)	<0.001
Persistent AF vs. non-AF	per unit	713/404	0.71 (0.60–0.84)	<0.001
	per SD	713/404	0.59 (0.45–0.77)	<0.001
Paroxysmal AF vs. persistent AF	per unit	813/409	0.62 (0.53–0.73)	<0.001
	per SD	813/409	0.48 (0.38–0.62)	<0.001

Values are adjusted odds ratios and 95% confidence intervals. Models were adjusted for available covariates used in the main multivariable analysis, including age, sex, smoking, drinking, hypertension, diabetes mellitus, coronary heart disease, LAD, LVEF, LVEDD, LVDD, SCr, ALT, AST, RBC, and PLT. .

### 3.4 Shape of the association between SPISE and AF

**Figure 2:**
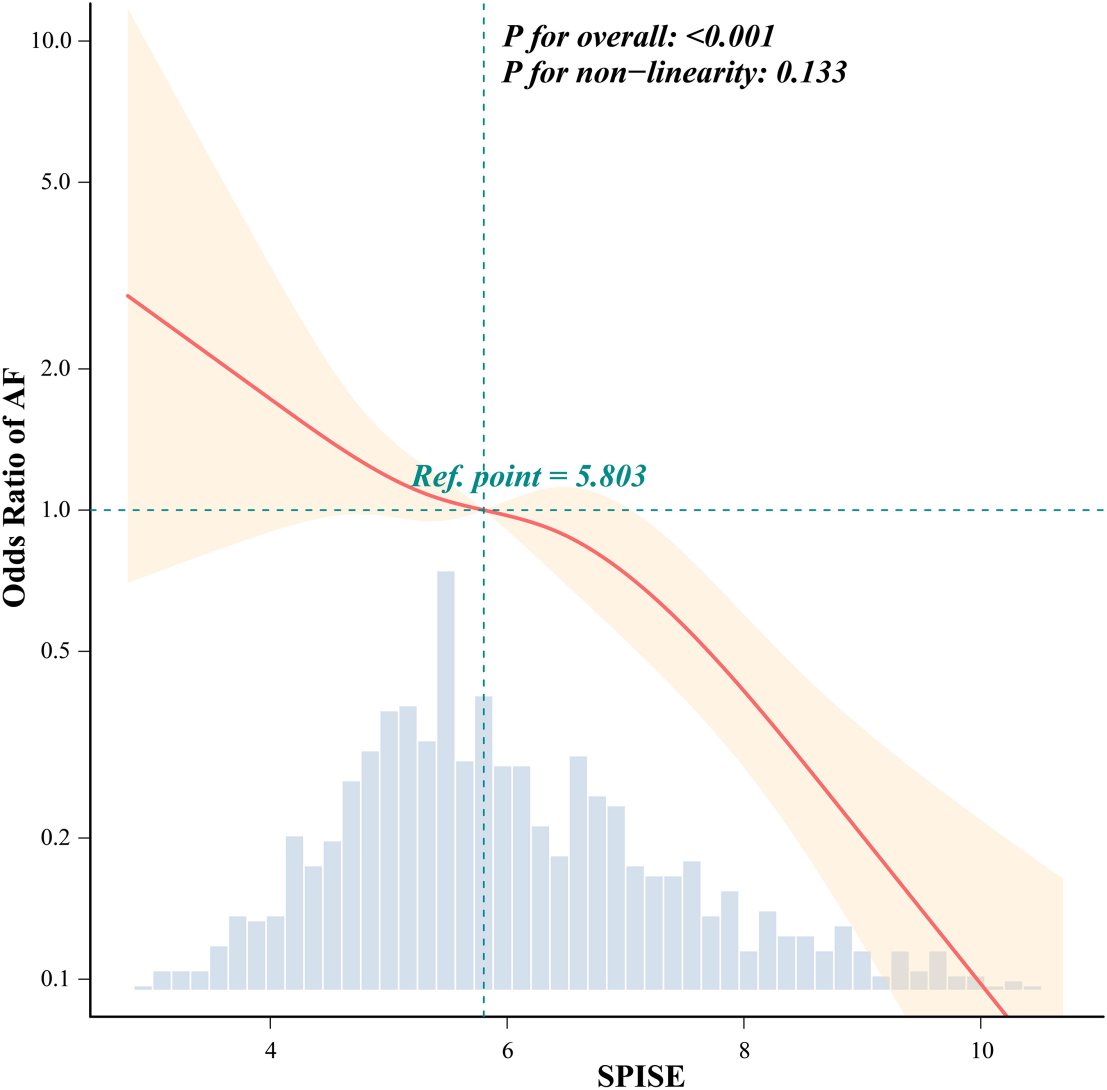
Restricted cubic spline analysis of the association between SPISE and atrial fibrillation. The spline model used four knots, and odds ratios were presented using SPISE = 5.803 as the reference value. The shaded area represents the 95% confidence interval.

**Figure 2** presents the spline-based analysis of SPISE in relation to AF. The overall association reached statistical significance (P for overall < 0.001), while no evidence supporting nonlinearity was found (P for non-linearity = 0.133). Higher SPISE values were accompanied by lower odds of AF over most of the observed range.

### 3.5 Candidate variable selection and determination of the AF classification model

**Figure 3:**
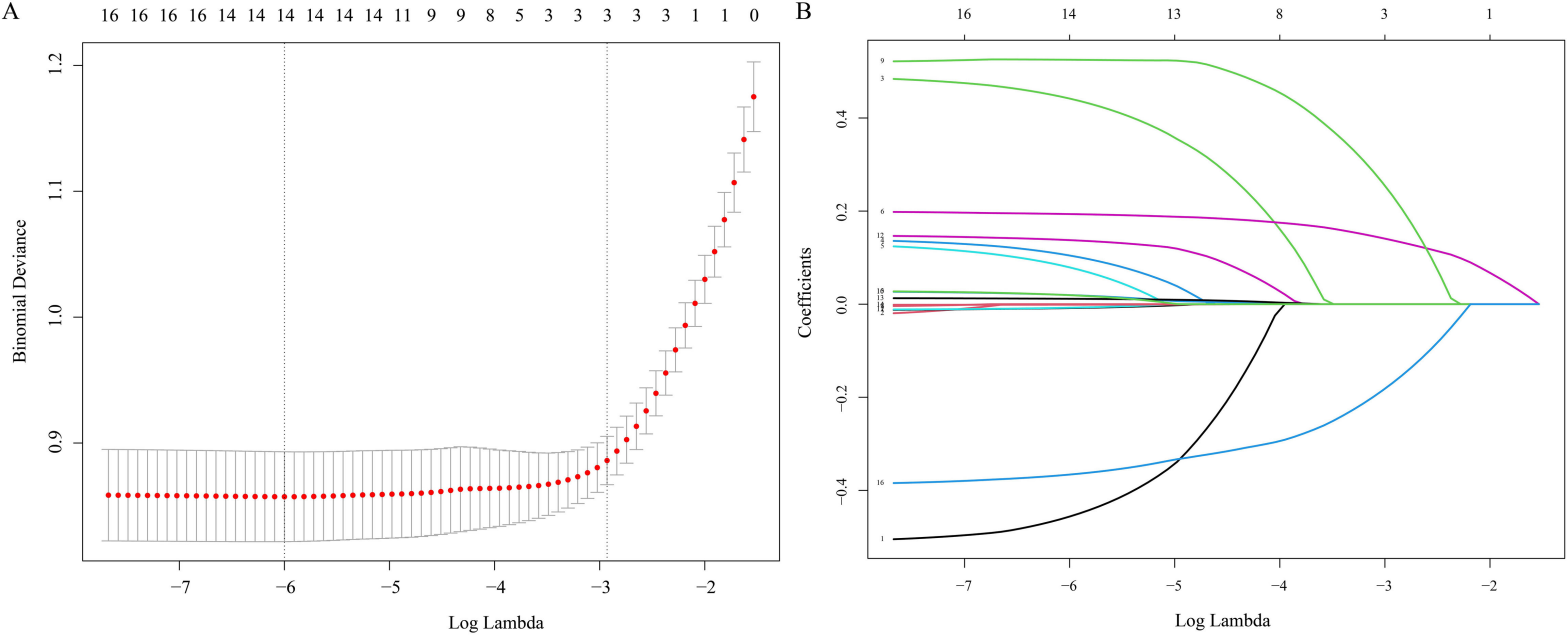
LASSO regression for candidate variable selection in the atrial fibrillation classification model. **(A)** Selection of the optimal penalty parameter (λ) by cross-validation; **(B)** LASSO coefficient profiles of candidate variables.

**Figure 3** outlines the preliminary reduction of candidate variables. According to the cross-validation results from the LASSO procedure, the penalty setting that retained a larger number of variables was adopted, and those variables were taken forward to the logistic regression analyses.

Univariable analysis was performed first, after which variables associated with AF were entered into the multivariable model (**Table 4**). The final AF classification model included SPISE, SCr, ALT, AST, LAD, LVDD, and DM. SPISE and ALT showed inverse associations with AF, whereas higher SCr, AST, and LAD, together with LVDD and DM, were related to greater odds of AF.

**Table 4:** Univariable and multivariable logistic regression analyses for atrial fibrillation.

Variables	Univariate analysis		Multivariate analysis	
	OR (95% CI)	P value	OR (95% CI)	P value
SPISE	0.64 (0.58 ~ 0.70)	<0.001	0.69 (0.62 ~ 0.77)	<0.001
PLT, per 50 × 109/L	0.87 (0.79 ~ 0.97)	0.015		
SCr, μmol/L	1.03 (1.02 ~ 1.04)	<0.001	1.01 (1.01 ~ 1.03)	0.020
ALT, U/L	1.01 (1.01 ~ 1.02)	0.009	0.99 (0.97 ~ 0.99)	0.038
AST, U/L	1.03 (1.01 ~ 1.05)	<0.001	1.03 (1.01 ~ 1.06)	0.012
LAD, mm	1.29 (1.25 ~ 1.34)	<0.001	1.23 (1.17 ~ 1.28)	<0.001
LVEF, %	0.91 (0.88 ~ 0.93)	<0.001		
LVEDD, mm	1.14 (1.11 ~ 1.18)	<0.001		
LVDD, n (%)		<0.001		0.011
No	1.00 (Reference)		1.00 (Reference)	
Yes	7.49 (5.49 ~ 10.22)		1.78 (1.14 ~ 2.77)	
Smoking, n (%)		0.036		
No	1.00 (Reference)			
Yes	0.62 (0.40 ~ 0.97)			
Hypertension, n (%)		<0.001		
No	1.00 (Reference)			
Yes	1.86 (1.42 ~ 2.43)			
DM, n (%)		<0.001		0.014
No	1.00 (Reference)		1.00 (Reference)	
Yes	2.19 (1.49 ~ 3.24)		1.77 (1.12 ~ 2.78)	
CHD, n (%)		0.103		
No	1.00 (Reference)			
Yes	1.32 (0.94 ~ 1.85)			
Stroke, n (%)		0.045		
No	1.00 (Reference)			
Yes	1.64 (1.01 ~ 2.67)			

ALT, alanine aminotransferase; AST, aspartate aminotransferase; CHD, coronary heart disease; DM, diabetes mellitus; LAD, left atrial diameter; LVDD, left ventricular diastolic dysfunction; LVEDD, left ventricular end-diastolic diameter; LVEF, left ventricular ejection fraction; SCr, Serum creatinine; PLT, platelet; SPISE, single point insulin sensitivity estimator. *P* - value less than 0.05 is expressed in bold.

### 3.6 Apparent performance of the final AF classification model

**Figure 4:**
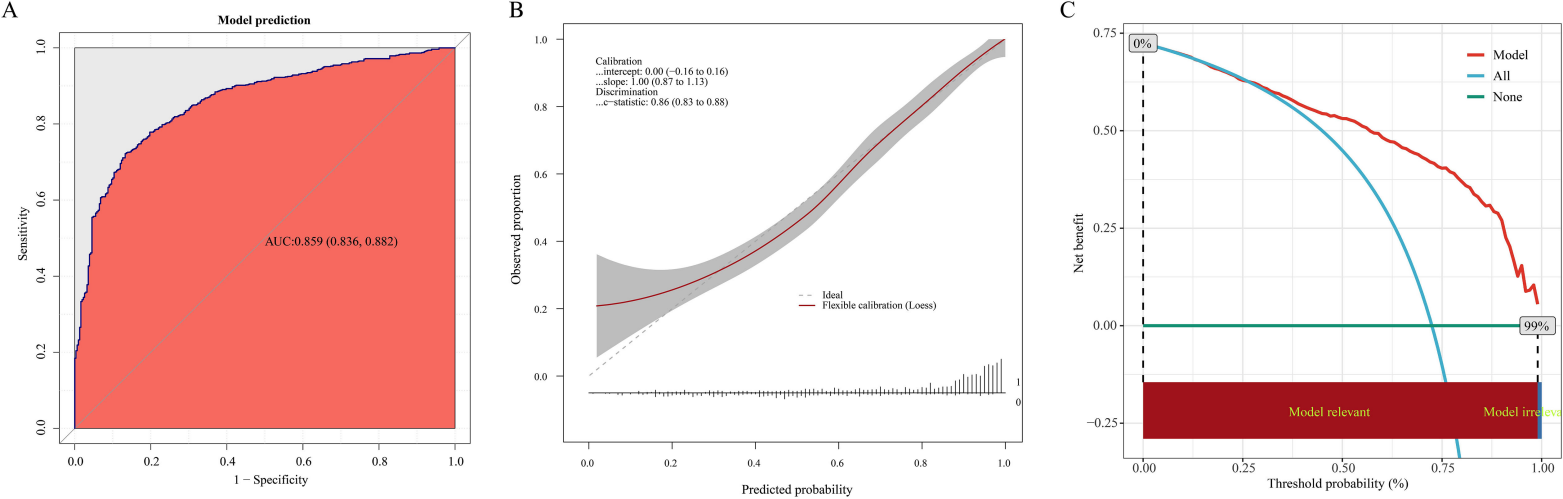
Apparent performance of the final atrial fibrillation classification model. **(A)** Receiver operating characteristic curve; **(B)** calibration curve; **(C)** decision-curve analysis.

An exploratory nomogram representing the fitted AF classification model is provided in **Supplementary Figure 1**. Apparent discrimination analysis yielded an AUC of 0.859 (95% CI: 0.836–0.882) (**Figure 4A**). Calibration metrics were 0.00 (-0.16 to 0.16) for the intercept and 1.00 (0.87–1.13) for the slope, and the calibration curve closely followed the reference line (**Figure 4B**). The apparent Brier score was 0.133 (**Table 5**).

**Table 5:** Internal validation of the final AF classification model.

Metric	Apparent performance	Repeated 10-fold cross-validation	Bootstrap optimism-corrected performance
AUC/C-statistic	0.859	0.855	0.854
Brier score	0.133	0.136	0.136
Calibration intercept	0.000	-0.001	–
Calibration slope	1.000	1.024	–
Emax	0.090	0.168	–
E90	0.031	0.110	–
Eavg	0.014	0.051	–
R2	0.437	0.409	–

The final AF classification model included SPISE, SCr, ALT, AST, LAD, LVDD, and DM. Repeated 10-fold cross-validation was performed for the specified final model. Bootstrap optimism correction was performed with 1000 resamples. These internal validation analyses did not repeat the full variable-selection process within each resampling iteration.AF, atrial fibrillation; ALT, alanine aminotransferase; AST, aspartate aminotransferase; AUC, area under the receiver operating characteristic curve; DM, diabetes mellitus; LAD, left atrial diameter; LVDD, left ventricular diastolic dysfunction; SCr, serum creatinine; SPISE, single-point insulin sensitivity estimator.

Decision-curve analysis showed that the model provided positive net benefit across a broad range of threshold probabilities (**Figure 4C**). The model curve was close to the “All” strategy at lower threshold probabilities and remained above the “None” strategy across the displayed range, supporting potential net benefit within this hospital-based analytic sample.

### 3.7 Internal validation of the final AF classification model

Internal validation metrics are summarized in **Table 5** and **Supplementary Figures 2**, **S3**. Across repeated 10-fold cross-validation, the mean C-statistic was 0.855 and the mean Brier score was 0.136. Mean values for the calibration intercept and slope were -0.001 and 1.024, respectively. Bootstrap optimism correction showed small optimism in model performance, with an optimism-corrected AUC of 0.854 and an optimism-corrected Brier score of 0.136.

The boxplots in **Supplementary Figure 2** show relatively narrow distributions for C (ROC) and the Brier score, whereas Emax varied more widely. In **Supplementary Figure 3**, the logistic calibration curve remained close to the ideal line, while the nonparametric curve lay above the ideal line at lower model-estimated probabilities and below it at higher model-estimated probabilities.

### 3.8 Incremental discriminative performance after adding SPISE to the reference models

**Figure 5:**
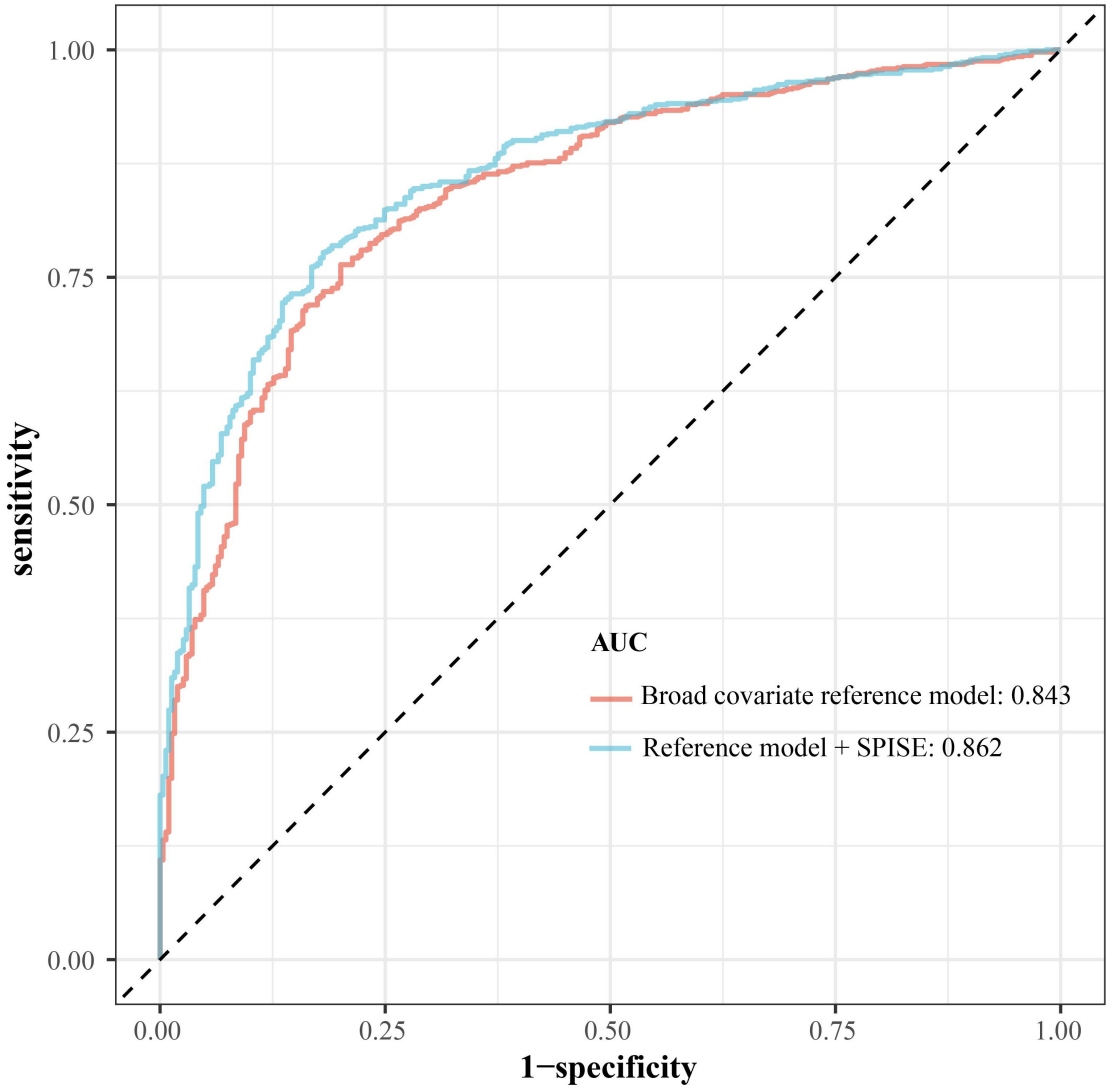
Incremental discrimination of SPISE beyond the broad covariate reference model for identifying atrial fibrillation.

With SPISE incorporated, the AUC of the broad covariate reference model increased from 0.843 (0.818–0.868) to 0.862 (0.839–0.885), corresponding to a statistically significant but numerically small absolute increase of 0.019 (P < 0.001; **Figure 5**).

Reclassification results are presented in **Table 6**. Category-free NRI was used because no established risk categories were available for identifying AF in this setting; therefore, no risk thresholds were applied. For the broad covariate reference model, addition of SPISE yielded a total NRI of 0.413 (0.285–0.542) and an IDI of 0.035 (0.020–0.050). For the LASSO-derived clinical reference model, addition of SPISE yielded a total NRI of 0.517 (0.390–0.644) and an IDI of 0.043 (0.028–0.059). All comparisons were significant at P < 0.001.

**Table 6:** Incremental discriminative value of SPISE beyond reference models for atrial fibrillation based on category-free NRI and IDI.

Model comparison	NRI (95% CI)	NRI *P* value	IDI (95% CI)	IDI *P* value
Broad covariate reference model	Ref		Ref	
Broad covariate reference model + SPISE	0.413 (0.285 - 0.542)	< 0.001	0.035 (0.020 - 0.050)	< 0.001
LASSO-derived clinical reference model	Ref		Ref	
LASSO-derived clinical reference model + SPISE	0.517 (0.390 - 0.644)	< 0.001	0.043 (0.028 - 0.059)	< 0.001

NRI was calculated as category-free NRI; no risk thresholds were used. The broad covariate reference model included sex, smoking, drinking, DM, hypertension, CHD, LAD, LVEF, LVEDD, LVDD, AST, ALT, SCr, PLT, and RBC. BMI, TG, and HDL-C were not included because they are mathematical components of SPISE. The LASSO-derived clinical reference model included the non-SPISE predictors retained in the final multivariable AF classification model: SCr, ALT, AST, LAD, LVDD, and DM.AF, atrial fibrillation; ALT, alanine aminotransferase; AST, aspartate aminotransferase; CHD, coronary heart disease; CI, confidence interval; DM, diabetes mellitus; IDI, integrated discrimination improvement; LAD, left atrial diameter; LVDD, left ventricular diastolic dysfunction; LVEDD, left ventricular end-diastolic diameter; LVEF, left ventricular ejection fraction; NRI, net reclassification improvement; PLT, platelet count; RBC, red blood cell count; SCr, serum creatinine; SPISE, single-point insulin sensitivity estimator.

### 3.9 Comparison of SPISE with its components and other insulin-resistance surrogate markers

SPISE was compared with two commonly used non-insulin-based insulin resistance surrogates, the TyG index and TG/HDL-C ratio. In the single-marker ROC analysis, TyG showed the highest AUC, followed by TG/HDL-C and SPISE. The AUCs were 0.717 (95% CI: 0.682–0.753) for TyG, 0.691 (95% CI: 0.654–0.727) for TG/HDL-C, and 0.678 (95% CI: 0.642–0.715) for SPISE (**Supplementary Figure 4**; **Supplementary Table 1**).

A model-based comparison was then performed by adding each index separately to the broad covariate reference model. The reference model had an AUC of 0.843 (95% CI: 0.818–0.868). The AUC increased to 0.862 (95% CI: 0.839–0.885) after adding SPISE, 0.885 (95% CI: 0.862–0.908) after adding TyG, and 0.868 (95% CI: 0.844–0.891) after adding TG/HDL-C. Thus, SPISE improved discrimination beyond the reference model, although TyG and TG/HDL-C showed numerically greater discrimination in these comparisons (**Supplementary Figure 5**; **Supplementary Table 2**).

To examine whether SPISE mainly reflected its component variables, we performed component-sensitivity analyses (**Supplementary Tables 3**, **S4**). SPISE showed the expected correlations with BMI, TG, and HDL-C. It was inversely correlated with BMI (Spearman ρ = -0.87) and TG (ρ = -0.61), and positively correlated with HDL-C (ρ = 0.37). Correlations with FBG (ρ = -0.27) and DM status (ρ = -0.11) were weaker. In the model including SPISE and the basic covariates, no substantial collinearity was observed, with a VIF of 1.20 for SPISE and a maximum VIF of 2.32. When SPISE and its components were entered simultaneously, VIFs increased for SPISE and BMI, consistent with the mathematical relationship between SPISE and its components. In the model comparison, adding SPISE improved the broad covariate reference model, whereas adding BMI, TG, and HDL-C separately showed slightly higher discrimination.

### 3.10 Robustness analyses addressing lipid-, liver-, and comorbidity-related confounding

Robustness analyses were performed to examine whether the SPISE–AF association was influenced by model specification, lipid- and glucose-related clinical status, cardiometabolic comorbidities, extreme lipid or liver-enzyme values, and other available proxies for lipid- or liver-metabolism–altering conditions. After removing RBC and PLT from the fully adjusted model, the association between SPISE and AF remained materially unchanged (OR per SD = 0.56, 95% CI: 0.47–0.67; P < 0.001; **Supplementary Table 5**). After additional adjustment for TC, LDL-C, and FBG, the association also remained stable (OR per SD = 0.56, 95% CI: 0.47–0.66; P < 0.001; **Supplementary Table 6**). Similar results were observed after excluding participants with CHD, stroke, or DM and after excluding participants with extreme TG, HDL-C, ALT, or AST values. Across the analyses addressing lipid/glucose adjustment, cardiovascular or metabolic comorbidity exclusion, and extreme laboratory values, the adjusted ORs per 1-SD increase in SPISE ranged from 0.51 to 0.56, and all remained statistically significant (**Supplementary Tables 7**-**S12**). In the E-value analysis, the E-value for the main fully adjusted per-SD association was 2.01. For the highest versus lowest SPISE quartile, the corresponding E-value was 2.72 (**Supplementary Table 13**).

The additional analyses focused on clinically relevant liver-enzyme abnormality, altered lipid status, extreme BMI, and broader cardiometabolic comorbidity burden also yielded consistent results. The fully adjusted ORs per 1-SD increase in SPISE were 0.56 (95% CI: 0.47–0.67) after excluding participants with ALT or AST >80 U/L, 0.55 (95% CI: 0.46–0.66) after excluding participants with ALT or AST >80 U/L or extreme TG/HDL-C values, 0.60 (95% CI: 0.50–0.72) after excluding participants with LDL-C <1.8 mmol/L, 0.56 (95% CI: 0.47–0.66) after excluding participants with extreme BMI values, and 0.60 (95% CI: 0.44–0.82) after excluding participants with CHD, stroke, DM, or hypertension. All associations remained statistically significant (**Supplementary Tables 14**-**S18**).

## 4 Discussion

The present analysis evaluated the relationship between SPISE and AF in a hospital-based population. Three main findings emerged. First, lower SPISE remained associated with AF after adjustment for available clinical, laboratory, and echocardiographic covariates. Second, the association was largely inverse and approximately linear. Third, adding SPISE to the reference models produced statistically significant improvements in discrimination and reclassification for AF status, although the absolute gain in AUC was small. These findings suggest that SPISE captures metabolic information related to AF status in this hospital-based dataset, while its clinical role should be interpreted cautiously.

A notable pattern in the baseline comparison was that SPISE was lowest among participants with paroxysmal AF rather than persistent AF. This finding does not necessarily indicate that paroxysmal AF is metabolically more severe than persistent AF. In a hospital-based cross-sectional dataset, AF subtype may reflect differences in presentation, referral, previous treatment, and cardiac structure, rather than a simple sequence of disease progression. These subtype-specific findings should be regarded as exploratory, while the main interpretation of the study remains focused on the overall association between SPISE and AF status.

The overall direction of these findings is consistent with previous observational work linking insulin resistance to AF (9). Earlier epidemiological studies have reported that insulin resistance is associated with AF (11, 22, 23). More recently, increasing attention has been given to non-insulin-based metabolic indices in AF research (24). Indices such as TyG, METS-IR, and their derivatives have been examined in relation to AF occurrence or post-ablation recurrence (25–27), indicating that simplified markers derived from routine metabolic variables may capture part of the metabolic profile associated with AF. Against this background, the current study extends that line of work to SPISE and shows an association with AF after multivariable adjustment, as well as incremental information beyond available reference models. In that sense, the present findings add evidence for SPISE as a potential adjunctive marker for AF-related metabolic phenotyping, rather than as a marker for predicting incident AF.

At the same time, these results should not be interpreted as establishing causality. Mendelian randomization studies have not consistently supported a stable causal relationship between genetically predicted insulin resistance and AF (12). The present results therefore support an association between metabolic phenotype and AF status, rather than a direct causal pathway. Because SPISE and AF status were assessed cross-sectionally, this analysis cannot determine whether lower SPISE preceded AF onset or reflected clinical and metabolic changes accompanying AF (28).

The quartile results should be viewed in the context of the continuous and spline-based analyses. The quartile findings do not indicate a clear biological cutoff for SPISE. In the present study, the continuous model, per-SD model, ordinal trend test, and restricted cubic spline analysis together supported an overall inverse association rather than a discrete threshold effect. Thus, SPISE should currently be interpreted as a continuous metabolic marker rather than as an index with an established clinical cutoff for AF assessment.

There is also biological plausibility for the observed association. Reduced insulin sensitivity is a common feature of obesity and diabetes, both of which are closely related to AF (29). Previous experimental and clinical studies have linked impaired insulin sensitivity to lipid accumulation, chronic low-grade inflammation, oxidative stress, and fibrotic remodeling, all of which are relevant to atrial structural change (30–33). Impaired insulin signaling may additionally affect ion channel function and intracellular calcium handling, thereby contributing to electrical remodeling (34). These pathways may help explain why lower SPISE was observed among participants with AF in the present study. However, they should be viewed as possible biological explanations for the observed cross-sectional association, rather than as causal evidence from the present analysis.

Because SPISE is calculated from BMI, TG, and HDL-C, its association with AF should not be interpreted as independent of these variables. The component-sensitivity analyses confirmed the expected overlap, especially with BMI and TG. This does not negate the usefulness of SPISE, but it refines its interpretation: SPISE is best viewed as a concise insulin-sensitivity surrogate that integrates routinely available anthropometric and lipid measurements into a single index.

The comparison with TyG and TG/HDL-C further refined the interpretation of SPISE. TyG and TG/HDL-C showed numerically higher single-marker AUCs than SPISE, and TyG also showed the highest AUC when added to the broad covariate reference model. Therefore, SPISE should not be interpreted as superior to these established indices. Rather, SPISE may be viewed as a complementary metabolic marker that integrates BMI, TG, and HDL-C and provides additional information beyond clinical covariates in this hospital-based sample. One possible advantage of TyG is that it directly incorporates fasting glucose and triglycerides, which may explain its stronger discrimination in the present dataset. By contrast, SPISE does not include fasting glucose and may therefore capture a different metabolic construct, with greater emphasis on adiposity- and lipid-related insulin sensitivity. This feature may be useful when the aim is to summarize an adiposity-lipid metabolic phenotype or when insulin measurements are unavailable. However, SPISE also has limitations: because it is derived from BMI, TG, and HDL-C, it may be influenced by body-size distribution, lipid-altering treatment, and other conditions affecting lipid metabolism. These considerations suggest that SPISE should be interpreted as a complementary marker rather than a replacement for TyG, TG/HDL-C, or other commonly used metabolic indices.

From a practical standpoint, SPISE is appealing because it can be calculated from routinely available BMI and lipid measurements and does not require insulin testing. In the present study, adding SPISE to the reference models improved AUC, NRI, and IDI. The clinical meaning of these improvements, however, should be interpreted cautiously. The absolute AUC increase was only 0.019, indicating that the additional discriminative gain was limited despite statistical significance. Similarly, the NRI and IDI results indicate improved model separation in the present dataset, but they do not demonstrate that SPISE would change clinical decisions, improve AF detection, or affect patient outcomes. Therefore, SPISE should not be presented as a screening or diagnostic tool for AF. A more appropriate interpretation is that SPISE may help describe the metabolic phenotype of patients evaluated for AF and may provide a simple adjunctive marker in research settings where insulin measurements are unavailable. AF identification in clinical practice remains dependent on electrocardiography, rhythm monitoring, and comprehensive clinical evaluation. Prospective external validation is needed before SPISE can be considered for routine AF-related assessment.

Several limitations should be acknowledged. The study was single-center, retrospective, and cross-sectional, which precludes inference about temporal sequence, causality, or incident AF prediction. AF ascertainment relied on clinical data and routine evaluation, so asymptomatic or brief paroxysmal episodes may have been missed, introducing potential outcome misclassification. Although multiple available clinical, laboratory, and echocardiographic variables were adjusted for, residual confounding cannot be excluded. Information on medication use was not available in the released dataset, including lipid-lowering therapy, specific antihypertensive agents, and glucose-lowering treatment. This limitation is particularly relevant to the present analysis because SPISE is calculated from BMI and lipid parameters. Lipid-lowering therapies may directly modify TG and HDL-C concentrations and therefore influence SPISE values without necessarily reflecting the untreated metabolic or insulin-sensitivity status of an individual. In addition, antidiabetic therapies may affect fasting glucose, body weight, and the overall metabolic profile, whereas antihypertensive therapies may be associated with both AF risk and the severity of underlying cardiovascular disease. Therefore, unmeasured treatment patterns may have influenced both SPISE levels and AF status, leading to potential residual confounding or confounding by indication. The released dataset also did not contain separate information on liver cirrhosis, active malignancy, pregnancy, dietary supplement use, or other exposures that may alter lipid metabolism. These factors could not be directly excluded or adjusted for in the present secondary analysis.

To address this issue as far as possible with the available data, several robustness analyses were performed, including additional adjustment for TC, LDL-C, and FBG; exclusion of participants with CHD, stroke, or DM; exclusion of participants with extreme TG, HDL-C, ALT, or AST values; exclusion of participants with clinically elevated liver enzymes; exclusion of participants with clinically elevated liver enzymes or extreme TG/HDL-C values; exclusion of participants with very low LDL-C levels; exclusion of participants with extreme BMI values; and exclusion of participants with CHD, stroke, DM, or hypertension. The association between SPISE and AF remained materially consistent across these analyses. Nevertheless, these indirect analyses cannot replace direct information on medication exposure, treatment duration, treatment intensity, or treatment indication. Accordingly, the present findings should be interpreted as associations observed after adjustment for available clinical and laboratory variables, rather than as estimates fully independent of medication-related influences. Future studies with detailed medication records are needed to determine whether the SPISE–AF association persists after accounting for lipid-lowering, antidiabetic, and antihypertensive therapies. Information on obstructive sleep apnea and physical activity was also unavailable, and residual confounding from these factors remains possible.

In addition, the study population was hospital-based, and the non-AF group consisted of hospitalized participants without documented AF rather than healthy community controls. The final analytic sample included 813 participants with AF and 309 without AF, corresponding to an AF proportion of 72.5%. This case distribution is substantially higher than would be expected in an unselected hospital or community screening population and may introduce selection bias and spectrum bias. Primary hospitalization diagnoses for the non-AF participants were not available in the released dataset. Details of the sampling framework, including whether the source cohort was consecutively assembled, were also unavailable. Therefore, we could not determine whether the high AF proportion reflected the original sampling design, referral patterns, or other features of the hospital-based cohort.

The nomogram was retained only as an exploratory visual representation of the fitted AF classification model and should not be interpreted as a clinically ready prediction instrument. Although repeated 10-fold cross-validation and bootstrap optimism correction were performed, no independent external validation dataset was available. Therefore, the reported performance should be interpreted as internal performance within the present analytic sample, and external validation remains necessary before the model can be considered for use in other clinical settings.

Despite these limitations, the study adds clinically relevant information. In contrast to earlier work centered mainly on traditional surrogate markers of insulin resistance, the present analysis introduces SPISE into the AF field and evaluates its association with AF, the shape of that association, and its incremental information beyond available reference models. Further work in prospective cohorts will be needed to determine whether SPISE is related to incident AF. Future studies may also examine whether SPISE helps characterize metabolic heterogeneity among patients with AF, including recurrence after ablation or thromboembolic outcomes.

## 5 Conclusion

In this hospital-based cross-sectional population, lower SPISE was associated with AF after multivariable adjustment, and the association was characterized primarily by an approximately linear inverse pattern. Incorporating SPISE into the reference models improved model discrimination and reclassification for AF status; however, the absolute improvement in discrimination was small. These findings support the potential value of SPISE as an adjunctive marker for AF-related metabolic phenotyping, but they do not establish SPISE as a stand-alone tool for routine AF assessment. Prospective studies are needed to determine whether SPISE is related to incident AF and whether it provides clinically actionable information beyond established AF assessment strategies.

## Data Availability

The original contributions presented in the study are included in the article/**Supplementary Material**. Further inquiries can be directed to the corresponding author/s.
