# Supplementary material for: Single-point insulin sensitivity estimator and atrial fibrillation: association and incremental discriminative value in a hospital-based population
Source: Front Endocrinol (Lausanne). 2026 Jul 1;17:1862319. doi: 10.3389/fendo.2026.1862319 (PMC13368470; doi:10.3389/fendo.2026.1862319)
Supplement: Supplementary file 1 [file DataSheet1.pdf]

Supplementary Material

Supplementary Table S1. Single-marker ROC comparison of SPISE, TyG index, and TG/HDL-C ratio for identifying atrial fibrillation.

Supplementary Table S2. Model-based ROC comparison after adding SPISE, TyG index, or TG/HDL-C ratio to the broad covariate reference model.

Supplementary Table S3. Correlations and collinearity diagnostics involving SPISE.

Supplementary Table S4. Component-sensitivity comparison using the broad covariate reference model.

Supplementary Table S5. Sensitivity analysis of the association between SPISE and atrial fibrillation after excluding RBC and PLT from the fully adjusted model (n=1122).

Supplementary Table S6. Association between SPISE and AF after additional adjustment for TC, LDL-C, and FBG (n=1122).

Supplementary Table S7. Sensitivity analysis excluding participants with CHD (n=890).

Supplementary Table S8. Sensitivity analysis excluding participants with CHD or stroke (n=803).

Supplementary Table S9. Sensitivity analysis excluding participants with CHD, stroke, or DM (n=675).

Supplementary Table S10. Sensitivity analysis excluding participants with extreme TG values (n=1109).

Supplementary Table S11. Sensitivity analysis excluding participants with extreme HDL-C values (n=1106).

Supplementary Table S12. Sensitivity analysis excluding participants with extreme ALT or AST values (n=1108).

Supplementary Table S13. E-value analysis for associations between SPISE and AF in logistic regression models (n=1122).

Supplementary Table S14. Sensitivity analysis after excluding participants with clinically elevated liver enzymes defined as ALT or AST >80 U/L (n=1096).

Supplementary Table S15. Sensitivity analysis after excluding participants with clinically elevated liver enzymes or extreme TG/HDL-C values (n=1062).

Supplementary Table S16. Sensitivity analysis after excluding participants with very low LDL-C levels defined as LDL-C <1.8 mmol/L (n=1012).

Supplementary Table S17. Sensitivity analysis after excluding participants with extreme BMI values (n=1098).

Supplementary Table S18. Sensitivity analysis after excluding participants with CHD, stroke, DM, or hypertension (n=421).

Supplementary Figure S1. Exploratory graphical representation of the fitted AF classification model.

Supplementary Figure S2. Distribution of model performance metrics across repeated k-fold cross-validation.

Supplementary Figure S3. Calibration curve of the final model in internal cross-validation.

Supplementary Figure S4. Receiver operating characteristic curves comparing SPISE, TyG index, and TG/HDL-C ratio for identifying atrial fibrillation.

Supplementary Figure S5. Model-based ROC comparison after adding SPISE, TyG index, or TG/HDL-C ratio to the broad covariate reference model.

Supplementary Table S1. Single-marker ROC comparison of SPISE, TyG index, and TG/HDL-C ratio for identifying atrial fibrillation

Marker	AUC (95% CI)	Optimal cutoff	Sensitivity	Specificity
SPISE	0.678 (0.642–0.715)	6.567	0.752	0.537
TyG index	0.717 (0.682–0.753)	8.449	0.856	0.511
TG/HDL-C ratio	0.691 (0.654–0.727)	2.282	0.828	0.498

Notes:
Optimal cutoff values were determined using the Youden index.
Abbreviations: AF, atrial fibrillation; AUC, area under the receiver operating characteristic curve; HDL-C, high-density lipoprotein cholesterol; ROC, receiver operating characteristic; SPISE, single-point insulin sensitivity estimator; TG, triglyceride; TyG, triglyceride-glucose.

Supplementary Table S2. Model-based ROC comparison after adding SPISE, TyG index, or TG/HDL-C ratio to the broad covariate reference model

Model	Added marker	AUC (95% CI)	ΔAUC vs reference	Optimal cutoff	Sensitivity	Specificity	Accuracy
Broad covariate reference model	None	0.843 (0.818–0.868)	Ref	0.710	0.760	0.800	0.770
Reference model + SPISE	SPISE	0.862 (0.839–0.885)	0.019	0.710	0.780	0.820	0.790
Reference model + TyG index	TyG index	0.885 (0.862–0.908)	0.042	0.720	0.800	0.850	0.820
Reference model + TG/HDL-C ratio	TG/HDL-C ratio	0.868 (0.844–0.891)	0.025	0.730	0.770	0.850	0.790

Notes:
The broad covariate reference model included sex, smoking, drinking, DM, hypertension, CHD, LAD, LVEF, LVEDD, LVDD, AST, ALT, SCr, PLT, and RBC.
ΔAUC was calculated relative to the broad covariate reference model. Optimal cutoff values were determined using the Youden index.
Abbreviations: AF, atrial fibrillation; ALT, alanine aminotransferase; AST, aspartate aminotransferase; AUC, area under the receiver operating characteristic curve; CHD, coronary heart disease; CI, confidence interval; DM, diabetes mellitus; HDL-C, high-density lipoprotein cholesterol; LAD, left atrial diameter; LVDD, left ventricular diastolic dysfunction; LVEDD, left ventricular end-diastolic diameter; LVEF, left ventricular ejection fraction; PLT, platelet count; RBC, red blood cell count; ROC, receiver operating characteristic; SCr, serum creatinine; SPISE, single-point insulin sensitivity estimator; TG, triglyceride; TyG, triglyceride-glucose.

Supplementary Table S3. Correlations and collinearity diagnostics involving SPISE

Analysis	Variable / item	Estimate	P value / note
Spearman correlation with SPISE	BMI	-0.87	<0.001
	TG	-0.61	<0.001
	HDL-C	0.37	<0.001
	FBG	-0.27	<0.001
	DM status	-0.11	<0.001
VIF in main model	SPISE	1.20	Main model included SPISE but not BMI, TG, or HDL-C
	Maximum VIF	2.32	No substantial collinearity observed
VIF when SPISE and components were entered simultaneously	SPISE	9.39	Expected overlap with component variables
	BMI	6.76	Expected overlap with SPISE
	TG	2.03	

HDL-C	1.49

Notes: Spearman correlations were calculated between SPISE and the indicated variables. DM status was coded as a binary variable.
The main VIF model included SPISE and the available covariates used in the main multivariable analysis but did not include BMI, TG, or HDL-C.
The simultaneous model included SPISE together with BMI, TG, and HDL-C to assess expected mathematical overlap; it was not used for coefficient interpretation.
Abbreviations: BMI, body mass index; DM, diabetes mellitus; FBG, fasting blood glucose; HDL-C, high-density lipoprotein cholesterol; SPISE, single-point insulin sensitivity estimator; TG, triglycerides; VIF, variance inflation factor.

Supplementary Table S4. Component-sensitivity comparison using the broad covariate reference model

Model	Added variables	AUC	Brier score	Interpretation
Broad covariate reference model	None	0.843	0.138	Base model for the component-sensitivity analysis
Broad covariate reference model + SPISE	SPISE	0.862	0.131	Adding SPISE improved discrimination and Brier score
Broad covariate reference model + BMI + TG + HDL-C	BMI, TG, HDL-C	0.871	0.124	Component-based model showed slightly higher performance
Broad covariate reference model + BMI + TG + HDL-C + SPISE	BMI, TG, HDL-C, SPISE	0.881	0.120	Exploratory only; not used for coefficient interpretation because of expected overlap

Notes:
This sensitivity analysis was performed to compare SPISE with its component variables; it does not replace the final AF classification model reported in the main manuscript.
The broad covariate reference model included sex, smoking, drinking, diabetes mellitus, hypertension, coronary heart disease, LAD, LVEF, LVEDD, LVDD, AST, ALT, SCr, PLT, and RBC.
The model including SPISE and all component variables simultaneously was used only to evaluate overlap and performance, not to interpret individual coefficients.
Abbreviations: AUC, area under the receiver operating characteristic curve; BMI, body mass index; HDL-C, high-density lipoprotein cholesterol; LAD, left atrial diameter; LVEF, left ventricular ejection fraction; LVEDD, left ventricular end-diastolic diameter; LVDD, left ventricular diastolic dysfunction; PLT, platelet; RBC, red blood cell; SCr, serum creatinine; SPISE, single-point insulin sensitivity estimator;

TG, triglycerides.

Supplementary Table S5. Sensitivity analysis of the association between SPISE and atrial fibrillation after excluding RBC and PLT from the fully adjusted model (n=1122).

Characteristic	Model1		Model2		Model3	
	OR(95% CI)	*P*-value	OR(95% CI)	*P*-value	OR(95% CI)	*P*-value
Continuous SPISE (per unit)	0.64 (0.58~0.70)	<0.001	0.69 (0.62~0.77)	<0.001	0.69 (0.61~0.77)	<0.001
Continuous SPISE (per SD)	0.51 (0.44~0.58)	<0.001	0.57 (0.48~0.67)	<0.001	0.56 (0.47~0.67)	<0.001
Q1	Ref.		Ref.		Ref.	
Q2	1.16 (0.75~1.79)	0.517	1.39 (0.85~2.28)	0.193	1.40 (0.84~2.32)	0.192
Q3	0.61 (0.41~0.91)	0.015	0.76 (0.48~1.21)	0.243	0.74 (0.46~1.20)	0.222
Q4	0.25 (0.17~0.36)	<0.001	0.36 (0.23~0.55)	<0.001	0.37 (0.23~0.59)	<0.001
P for trend	0.59 (0.52~0.67)	<0.001	0.67 (0.58~0.77)	<0.001	0.68 (0.58~0.79)	<0.001

Model 1: Unadjusted.
Model 2: Adjusted for age, sex, and LAD.
Model 3: Adjusted for age, sex, smoking, hypertension, drinking, DM, CHD, LAD, LVEF, LVEDD, LVDD, Scr, ALT, AST.
Abbreviations: AF, atrial fibrillation; ALT, alanine aminotransferase; AST, aspartate aminotransferase; CHD, coronary heart disease; CI, confidence interval; DM, diabetes mellitus; LAD, left atrial diameter; LVDD, left ventricular diastolic

dysfunction; LVEDD, left ventricular end-diastolic diameter; LVEF, left ventricular ejection fraction; OR, odds ratio; SCr, serum creatinine; SPISE, single-point insulin sensitivity estimator.

Supplementary Table S6. Association between SPISE and AF after additional adjustment for TC, LDL-C, and FBG (n=1122).

Characteristic	Model1		Model2		Model3	
	OR(95% CI)	*P*-value	OR(95% CI)	*P*-value	OR(95% CI)	*P*-value
Continuous SPISE (per unit)	0.64 (0.58~0.70)	<0.001	0.69 (0.62~0.77)	<0.001	0.68 (0.61~0.76)	<0.001
Continuous SPISE (per SD)	0.51 (0.44~0.58)	<0.001	0.57 (0.48~0.67)	<0.001	0.56 (0.47~0.66)	<0.001
Q1	Ref.		Ref.		Ref.	
Q2	1.16 (0.75~1.79)	0.517	1.39 (0.85~2.28)	0.193	1.41 (0.85~2.36)	0.183
Q3	0.61 (0.41~0.91)	0.015	0.76 (0.48~1.21)	0.243	0.73 (0.45~1.19)	0.210
Q4	0.25 (0.17~0.36)	<0.001	0.36 (0.23~0.55)	<0.001	0.36 (0.23~0.58)	<0.001
P for trend	0.59 (0.52~0.67)	<0.001	0.67 (0.58~0.77)	<0.001	0.67 (0.58~0.79)	<0.001

Model 1: Unadjusted.
Model 2: Adjusted for age, sex, and LAD.
Model 3: Adjusted for age, sex, smoking, hypertension, drinking, DM, CHD, LAD, LVEF, LVEDD, LVDD, Scr, ALT, AST, PLT, RBC, TC, LDL-C and FBG.
Abbreviations: AF, atrial fibrillation; ALT, alanine aminotransferase; AST, aspartate aminotransferase; CHD, coronary heart disease; CI, confidence interval; DM, diabetes mellitus; FBG, fasting blood glucose; LAD, left atrial diameter; LDL-

C, low-density lipoprotein cholesterol; LVDD, left ventricular diastolic dysfunction; LVEDD, left ventricular end-diastolic diameter; LVEF, left ventricular ejection fraction; OR, odds ratio; PLT, platelet count; RBC, red blood cell count; SCr, serum creatinine; SPISE, single-point insulin sensitivity estimator; TC, total cholesterol.

Supplementary Table S7. Sensitivity analysis excluding participants with CHD (n=890).

Characteristic	Model1		Model2		Model3	
	OR(95% CI)	*P*-value	OR(95% CI)	*P*-value	OR(95% CI)	*P*-value
Continuous SPISE (per unit)	0.65 (0.59~0.72)	<0.001	0.70 (0.62~0.79)	<0.001	0.68 (0.60~0.77)	<0.001
Continuous SPISE (per SD)	0.52 (0.45~0.61)	<0.001	0.58 (0.48~0.69)	<0.001	0.56 (0.46~0.68)	<0.001
Q1	Ref.		Ref.		Ref.	
Q2	1.09 (0.68~1.74)	0.735	1.30 (0.76~2.23)	0.345	1.32 (0.76~2.30)	0.324
Q3	0.63 (0.41~0.99)	0.043	0.82 (0.49~1.36)	0.437	0.77 (0.45~1.31)	0.334
Q4	0.26 (0.17~0.40)	<0.001	0.36 (0.22~0.58)	<0.001	0.35 (0.20~0.59)	<0.001
P for trend	0.61 (0.53~0.70)	<0.001	0.68 (0.58~0.80)	<0.001	0.67 (0.57~0.80)	<0.001

Model 1: Unadjusted.
Model 2: Adjusted for age, sex, and LAD.
Model 3: Adjusted for age, sex, smoking, hypertension, drinking, DM, LAD, LVEF, LVEDD, LVDD, Scr, ALT, AST, PLT, RBC.
Abbreviations: AF, atrial fibrillation; ALT, alanine aminotransferase; AST, aspartate aminotransferase; CHD, coronary heart disease; CI, confidence interval; DM, diabetes mellitus; LAD, left atrial diameter; LVDD, left ventricular diastolic dysfunction; LVEDD, left ventricular end-diastolic diameter; LVEF, left ventricular ejection fraction; OR, odds ratio; PLT,

platelet count; RBC, red blood cell count; SCr, serum creatinine; SPISE, single-point insulin sensitivity estimator.

Supplementary Table S8. Sensitivity analysis excluding participants with CHD or stroke (n=803).

Characteristic	Model1		Model2		Model3	
	OR(95% CI)	*P*-value	OR(95% CI)	*P*-value	OR(95% CI)	*P*-value
Continuous SPISE (per unit)	0.64 (0.58~0.72)	<0.001	0.70 (0.62~0.79)	<0.001	0.68 (0.59~0.78)	<0.001
Continuous SPISE (per SD)	0.51 (0.43~0.60)	<0.001	0.58 (0.48~0.70)	<0.001	0.55 (0.45~0.68)	<0.001
Q1	Ref.		Ref.		Ref.	
Q2	1.17 (0.71~1.92)	0.542	1.38 (0.78~2.43)	0.267	1.44 (0.80~2.58)	0.219
Q3	0.57 (0.36~0.91)	0.017	0.79 (0.46~1.34)	0.376	0.74 (0.43~1.29)	0.288
Q4	0.27 (0.18~0.43)	<0.001	0.40 (0.24~0.67)	<0.001	0.39 (0.23~0.68)	0.001
P for trend	0.62 (0.53~0.71)	<0.001	0.70 (0.60~0.83)	<0.001	0.69 (0.58~0.83)	<0.001

Model 1: Unadjusted.
Model 2: Adjusted for age, sex, and LAD.
Model 3: Adjusted for age, sex, smoking, hypertension, drinking, DM, LAD, LVEF, LVEDD, LVDD, Scr, ALT, AST, PLT, RBC.
Abbreviations: AF, atrial fibrillation; ALT, alanine aminotransferase; AST, aspartate aminotransferase; CHD, coronary heart disease; CI, confidence interval; DM, diabetes mellitus; LAD, left atrial diameter; LVDD, left ventricular diastolic dysfunction; LVEDD, left ventricular end-diastolic diameter; LVEF, left ventricular ejection fraction; OR, odds ratio; PLT,

platelet count; RBC, red blood cell count; SCr, serum creatinine; SPISE, single-point insulin sensitivity estimator.

Supplementary Table S9. Sensitivity analysis excluding participants with CHD, stroke, or DM (n=675).

Characteristic	Model1		Model2		Model3	
	OR(95% CI)	*P*-value	OR(95% CI)	*P*-value	OR(95% CI)	*P*-value
Continuous SPISE (per unit)	0.63 (0.56~0.71)	<0.001	0.68 (0.59~0.78)	<0.001	0.64 (0.55~0.75)	<0.001
Continuous SPISE (per SD)	0.50 (0.41~0.59)	<0.001	0.55 (0.45~0.68)	<0.001	0.51 (0.41~0.64)	<0.001
Q1	Ref.		Ref.		Ref.	
Q2	1.11 (0.65~1.88)	0.706	1.23 (0.68~2.23)	0.500	1.25 (0.67~2.31)	0.481
Q3	0.56 (0.34~0.91)	0.021	0.65 (0.37~1.14)	0.136	0.56 (0.31~1.02)	0.056
Q4	0.25 (0.15~0.40)	<0.001	0.33 (0.19~0.58)	<0.001	0.31 (0.17~0.56)	<0.001
P for trend	0.60 (0.51~0.70)	<0.001	0.66 (0.56~0.79)	<0.001	0.64 (0.53~0.78)	<0.001

Model 1: Unadjusted.
Model 2: Adjusted for age, sex, and LAD.
Model 3: Adjusted for age, sex, smoking, hypertension, drinking, LAD, LVEF, LVEDD, LVDD, SCr, ALT, AST, PLT, and RBC.
Abbreviations: AF, atrial fibrillation; ALT, alanine aminotransferase; AST, aspartate aminotransferase; CHD, coronary heart disease; CI, confidence interval; DM, diabetes mellitus; LAD, left atrial diameter; LVDD, left ventricular diastolic dysfunction; LVEDD, left ventricular end-diastolic diameter; LVEF, left ventricular ejection fraction; OR, odds ratio; PLT,

platelet count; RBC, red blood cell count; SCr, serum creatinine; SPISE, single-point insulin sensitivity estimator.

Supplementary Table S10. Sensitivity analysis excluding participants with extreme TG values (n=1109).

Characteristic	Model1		Model2		Model3	
	OR(95% CI)	*P*-value	OR(95% CI)	*P*-value	OR(95% CI)	*P*-value
Continuous SPISE (per unit)	0.64 (0.58~0.70)	<0.001	0.68 (0.61~0.76)	<0.001	0.67 (0.59~0.75)	<0.001
Continuous SPISE (per SD)	0.50 (0.44~0.58)	<0.001	0.56 (0.48~0.66)	<0.001	0.54 (0.46~0.65)	<0.001
Q1	Ref.		Ref.		Ref.	
Q2	1.14 (0.73~1.77)	0.573	1.38 (0.84~2.28)	0.207	1.39 (0.83~2.33)	0.210
Q3	0.57 (0.38~0.86)	0.007	0.71 (0.45~1.14)	0.154	0.67 (0.41~1.09)	0.103
Q4	0.24 (0.16~0.35)	<0.001	0.34 (0.22~0.54)	<0.001	0.34 (0.21~0.54)	<0.001
P for trend	0.58 (0.51~0.66)	<0.001	0.66 (0.57~0.76)	<0.001	0.66 (0.56~0.77)	<0.001

Extreme TG values were defined as values above the 99th percentile.
Model 1: Unadjusted.
Model 2: Adjusted for age, sex, and LAD.
Model 3: Adjusted for age, sex, smoking, hypertension, drinking, CHD, DM, LAD, LVEF, LVEDD, LVDD, SCr, ALT, AST, PLT, and RBC.
Abbreviations: AF, atrial fibrillation; ALT, alanine aminotransferase; AST, aspartate aminotransferase; CHD, coronary heart disease; CI, confidence interval; DM, diabetes mellitus; LAD, left atrial diameter; LVDD, left ventricular diastolic

dysfunction; LVEDD, left ventricular end-diastolic diameter; LVEF, left ventricular ejection fraction; OR, odds ratio; PLT, platelet count; RBC, red blood cell count; SCr, serum creatinine; SPISE, single-point insulin sensitivity estimator; TG, triglyceride.

Supplementary Table S11. Sensitivity analysis excluding participants with extreme HDL-C values (n=1106).

Characteristic	Model1		Model2		Model3	
	OR(95% CI)	*P*-value	OR(95% CI)	*P*-value	OR(95% CI)	*P*-value
Continuous SPISE (per unit)	0.64 (0.58~0.70)	<0.001	0.69 (0.62~0.77)	<0.001	0.68 (0.61~0.77)	<0.001
Continuous SPISE (per SD)	0.50 (0.44~0.58)	<0.001	0.57 (0.49~0.67)	<0.001	0.56 (0.47~0.67)	<0.001
Q1	Ref.		Ref.		Ref.	
Q2	1.16 (0.74~1.81)	0.509	1.37 (0.83~2.27)	0.216	1.41 (0.84~2.37)	0.190
Q3	0.54 (0.36~0.81)	0.003	0.69 (0.43~1.10)	0.118	0.68 (0.42~1.10)	0.117
Q4	0.25 (0.17~0.37)	<0.001	0.36 (0.23~0.56)	<0.001	0.37 (0.23~0.59)	<0.001
P for trend	0.59 (0.52~0.67)	<0.001	0.67 (0.58~0.77)	<0.001	0.67 (0.58~0.79)	<0.001

Extreme HDL-C values were defined as values below the 1st percentile or above the 99th percentile.
Model 1: Unadjusted.
Model 2: Adjusted for age, sex, and LAD.
Model 3: Adjusted for age, sex, smoking, hypertension, drinking, CHD, DM, LAD, LVEF, LVEDD, LVDD, SCr, ALT, AST, PLT, and RBC.
Abbreviations: AF, atrial fibrillation; ALT, alanine aminotransferase; AST, aspartate aminotransferase; CHD, coronary heart disease; CI, confidence interval; DM, diabetes mellitus; HDL-C, high-density lipoprotein cholesterol; LAD, left atrial

diameter; LVDD, left ventricular diastolic dysfunction; LVEDD, left ventricular end-diastolic diameter; LVEF, left ventricular ejection fraction; OR, odds ratio; PLT, platelet count; RBC, red blood cell count; SCr, serum creatinine; SPISE, single-point insulin sensitivity estimator.

Supplementary Table S12. Sensitivity analysis excluding participants with extreme ALT or AST values (n=1108).

Characteristic	Model1		Model2		Model3	
	OR(95% CI)	*P*-value	OR(95% CI)	*P*-value	OR(95% CI)	*P*-value
Continuous SPISE (per unit)	0.64 (0.58~0.70)	<0.001	0.69 (0.62~0.76)	<0.001	0.68 (0.61~0.77)	<0.001
Continuous SPISE (per SD)	0.50 (0.43~0.58)	<0.001	0.56 (0.48~0.66)	<0.001	0.56 (0.47~0.67)	<0.001
Q1	Ref.		Ref.		Ref.	
Q2	1.19 (0.77~1.86)	0.430	1.43 (0.86~2.36)	0.166	1.49 (0.89~2.50)	0.127
Q3	0.60 (0.40~0.89)	0.012	0.73 (0.46~1.17)	0.194	0.75 (0.46~1.23)	0.258
Q4	0.24 (0.16~0.35)	<0.001	0.35 (0.22~0.54)	<0.001	0.37 (0.23~0.60)	<0.001
P for trend	0.58 (0.51~0.66)	<0.001	0.66 (0.57~0.76)	<0.001	0.68 (0.58~0.79)	<0.001

Extreme liver-enzyme values were defined as ALT or AST values above the 99th percentile.
Model 1: Unadjusted.
Model 2: Adjusted for age, sex, and LAD.
Model 3: Adjusted for age, sex, smoking, hypertension, drinking, CHD, DM, LAD, LVEF, LVEDD, LVDD, SCr, ALT, AST, PLT, and RBC.
Abbreviations: AF, atrial fibrillation; ALT, alanine aminotransferase; AST, aspartate aminotransferase; CHD, coronary heart disease; CI, confidence interval; DM, diabetes mellitus; LAD, left atrial diameter; LVDD, left ventricular diastolic

dysfunction; LVEDD, left ventricular end-diastolic diameter; LVEF, left ventricular ejection fraction; OR, odds ratio; PLT, platelet count; RBC, red blood cell count; SCr, serum creatinine; SPISE, single-point insulin sensitivity estimator.

Supplementary Table S13. E-value analysis for associations between SPISE and AF in logistic regression models (n=1122).

Characteristic	Model1		Model2		Model3	
	OR(95% CI)	E-value	OR(95% CI)	E-value	OR(95% CI)	E-value
Continuous SPISE (per unit)	0.64 (0.58~0.70)	1.81	0.69 (0.62~0.77)	1.70	0.68 (0.61~0.76)	1.72
Continuous SPISE (per SD)	0.51 (0.44~0.58)	2.15	0.57 (0.48~0.67)	1.98	0.56 (0.47~0.66)	2.01
Q1	Ref.		Ref.		Ref.	
Q2	1.16 (0.75~1.79)	1.37	1.39 (0.85~2.28)	1.64	1.42 (0.86~2.36)	1.67
Q3	0.61 (0.41~0.91)	1.88	0.76 (0.48~1.21)	1.56	0.75 (0.46~1.21)	1.58
Q4	0.25 (0.17~0.36)	3.41	0.36 (0.23~0.55)	2.72	0.36 (0.23~0.58)	2.72
P for trend	0.59 (0.52~0.67)	1.93	0.67 (0.58~0.77)	1.74	0.68 (0.58~0.79)	1.72

E-values were calculated for the point estimates. Because AF was common in this hospital-based analytic sample, E-values were calculated after approximate conversion of odds ratios to the risk-ratio scale.
Model 1: Unadjusted.
Model 2: Adjusted for age, sex, and LAD.
Model 3: Adjusted for age, sex, smoking, hypertension, drinking, DM, CHD, LAD, LVEF, LVEDD, LVDD, SCr, ALT, AST, RBC, and PLT.

Abbreviations: AF, atrial fibrillation; ALT, alanine aminotransferase; AST, aspartate aminotransferase; CHD, coronary heart disease; CI, confidence interval; DM, diabetes mellitus; LAD, left atrial diameter; LVDD, left ventricular diastolic dysfunction; LVEDD, left ventricular end-diastolic diameter; LVEF, left ventricular ejection fraction; OR, odds ratio; PLT, platelet count; RBC, red blood cell count; SCr, serum creatinine; SPISE, single-point insulin sensitivity estimator.

Supplementary Table S14. Sensitivity analysis after excluding participants with clinically elevated liver enzymes defined as ALT or AST >80 U/L (n=1096).

Characteristic	Model1		Model2		Model3	
	OR(95% CI)	*P*-value	OR(95% CI)	*P*-value	OR(95% CI)	*P*-value
Continuous SPISE (per unit)	0.63 (0.58~0.69)	<0.001	0.68 (0.61~0.76)	<0.001	0.68 (0.61~0.77)	<0.001
Continuous SPISE (per SD)	0.49 (0.43~0.57)	<0.001	0.55 (0.47~0.65)	<0.001	0.56 (0.47~0.67)	<0.001
Q1	Ref.		Ref.		Ref.	
Q2	1.2 (0.77~1.88)	0.426	1.45 (0.87~2.40)	0.154	1.53 (0.91~2.58)	0.111
Q3	0.59 (0.39~0.89)	0.011	0.73 (0.46~1.16)	0.185	0.76 (0.47~1.25)	0.281
Q4	0.23 (0.16~0.34)	<0.001	0.33 (0.21~0.52)	<0.001	0.37 (0.23~0.61)	<0.001
P for trend	0.58 (0.51~0.66)	<0.001	0.65 (0.56~0.75)	<0.001	0.68 (0.58~0.79)	<0.001

Model 1: Unadjusted.
Model 2: Adjusted for age, sex, and LAD.
Model 3: Adjusted for age, sex, smoking, hypertension, drinking, DM, CHD, LAD, LVEF, LVEDD, LVDD, Scr, ALT, AST, PLT and RBC.
Abbreviations: AF, atrial fibrillation; ALT, alanine aminotransferase; AST, aspartate aminotransferase; CHD, coronary heart disease; CI, confidence interval; DM, diabetes mellitus; LAD, left atrial diameter; LVDD, left ventricular diastolic

dysfunction; LVEDD, left ventricular end-diastolic diameter; LVEF, left ventricular ejection fraction; OR, odds ratio; PLT, platelet count; RBC, red blood cell count; SCr, serum creatinine; SPISE, single-point insulin sensitivity estimator.

Supplementary Table S15. Sensitivity analysis after excluding participants with clinically elevated liver enzymes or extreme TG/HDL-C values (n=1062).

Characteristic	Model1		Model2		Model3	
	OR(95% CI)	*P*-value	OR(95% CI)	*P*-value	OR(95% CI)	*P*-value
Continuous SPISE (per unit)	0.63 (0.57~0.69)	<0.001	0.67 (0.60~0.75)	<0.001	0.67 (0.60~0.76)	<0.001
Continuous SPISE (per SD)	0.49 (0.43~0.57)	<0.001	0.55 (0.47~0.65)	<0.001	0.55 (0.46~0.66)	<0.001
Q1	Ref.		Ref.		Ref.	
Q2	1.23 (0.78~1.94)	0.366	1.49 (0.89~2.5)	0.128	1.54 (0.90~2.62)	0.113
Q3	0.57 (0.38~0.86)	0.007	0.71 (0.44~1.14)	0.161	0.71 (0.43~1.17)	0.178
Q4	0.24 (0.16~0.35)	<0.001	0.34 (0.21~0.53)	<0.001	0.36 (0.22~0.59)	<0.001
P for trend	0.58 (0.51~0.66)	<0.001	0.65 (0.56~0.75)	<0.001	0.66 (0.56~0.78)	<0.001

Clinically elevated liver enzymes were defined as ALT or AST >80 U/L. Extreme TG values were defined as values above the 99th percentile, and extreme HDL-C values were defined as values below the 1st percentile or above the 99th percentile.
Model 1: Unadjusted.
Model 2: Adjusted for age, sex, and LAD.
Model 3: Adjusted for age, sex, smoking, hypertension, drinking, DM, CHD, LAD, LVEF, LVEDD, LVDD, Scr, ALT, AST, PLT and RBC.

Abbreviations: AF, atrial fibrillation; ALT, alanine aminotransferase; AST, aspartate aminotransferase; CHD, coronary heart disease; CI, confidence interval; DM, diabetes mellitus; LAD, left atrial diameter; LVDD, left ventricular diastolic dysfunction; LVEDD, left ventricular end-diastolic diameter; LVEF, left ventricular ejection fraction; OR, odds ratio; PLT, platelet count; RBC, red blood cell count; SCr, serum creatinine; SPISE, single-point insulin sensitivity estimator.

Supplementary Table S16. Sensitivity analysis after excluding participants with very low LDL-C levels defined as LDL-C <1.8 mmol/L (n=1012).

Characteristic	Model1		Model2		Model3	
	OR(95% CI)	*P*-value	OR(95% CI)	*P*-value	OR(95% CI)	*P*-value
Continuous SPISE (per unit)	0.65 (0.59~0.72)	<0.001	0.72 (0.64~0.80)	<0.001	0.72 (0.64~0.80)	<0.001
Continuous SPISE (per SD)	0.52 (0.45~0.60)	<0.001	0.61 (0.51~0.72)	<0.001	0.60 (0.50~0.72)	<0.001
Q1	Ref.		Ref.		Ref.	
Q2	1.21 (0.76~1.93)	0.410	1.40 (0.83~2.37)	0.210	1.46 (0.85~2.51)	0.167
Q3	0.51 (0.34~0.77)	0.002	0.66 (0.41~1.07)	0.092	0.68 (0.41~1.12)	0.131
Q4	0.26 (0.17~0.38)	<0.001	0.39 (0.24~0.62)	<0.001	0.40 (0.24~0.66)	<0.001
P for trend	0.59 (0.52~0.68)	<0.001	0.68 (0.59~0.80)	<0.001	0.69 (0.59~0.82)	<0.001

Model 1: Unadjusted.
Model 2: Adjusted for age, sex, and LAD.
Model 3: Adjusted for age, sex, smoking, hypertension, drinking, DM, CHD, LAD, LVEF, LVEDD, LVDD, Scr, ALT, AST, PLT and RBC.
Abbreviations: AF, atrial fibrillation; ALT, alanine aminotransferase; AST, aspartate aminotransferase; CHD, coronary heart disease; CI, confidence interval; DM, diabetes mellitus; LAD, left atrial diameter; LVDD, left ventricular diastolic

dysfunction; LVEDD, left ventricular end-diastolic diameter; LVEF, left ventricular ejection fraction; OR, odds ratio; PLT, platelet count; RBC, red blood cell count; SCr, serum creatinine; SPISE, single-point insulin sensitivity estimator.

Supplementary Table S17. Sensitivity analysis after excluding participants with extreme BMI values (n=1098).

Characteristic	Model1		Model2		Model3	
	OR(95% CI)	*P*-value	OR(95% CI)	*P*-value	OR(95% CI)	*P*-value
Continuous SPISE (per unit)	0.61 (0.56~0.68)	<0.001	0.67 (0.60~0.75)	<0.001	0.66 (0.59~0.75)	<0.001
Continuous SPISE (per SD)	0.50 (0.43~0.58)	<0.001	0.56 (0.48~0.66)	<0.001	0.56 (0.47~0.66)	<0.001
Q1	Ref.		Ref.		Ref.	
Q2	1.19 (0.76~1.84)	0.445	1.45 (0.88~2.39)	0.143	1.48 (0.89~2.47)	0.134
Q3	0.61 (0.41~0.91)	0.015	0.77 (0.49~1.23)	0.277	0.76 (0.47~1.24)	0.271
Q4	0.26 (0.18~0.38)	<0.001	0.38 (0.24~0.59)	<0.001	0.39 (0.24~0.63)	<0.001
P for trend	0.60 (0.53~0.68)	<0.001	0.68 (0.59~0.79)	<0.001	0.69 (0.59~0.81)	<0.001

Extreme BMI values were defined as values below the 1st percentile or above the 99th percentile.
Model 1: Unadjusted.
Model 2: Adjusted for age, sex, and LAD.
Model 3: Adjusted for age, sex, smoking, hypertension, drinking, DM, CHD, LAD, LVEF, LVEDD, LVDD, Scr, ALT, AST, PLT and RBC.
Abbreviations: AF, atrial fibrillation; ALT, alanine aminotransferase; AST, aspartate aminotransferase; CHD, coronary heart disease; CI, confidence interval; DM, diabetes mellitus; LAD, left atrial diameter; LVDD, left ventricular diastolic

dysfunction; LVEDD, left ventricular end-diastolic diameter; LVEF, left ventricular ejection fraction; OR, odds ratio; PLT, platelet count; RBC, red blood cell count; SCr, serum creatinine; SPISE, single-point insulin sensitivity estimator.

Supplementary Table S18. Sensitivity analysis after excluding participants with CHD, stroke, DM, or hypertension (n=421).

Characteristic	Model1		Model2		Model3	
	OR(95% CI)	*P*-value	OR(95% CI)	*P*-value	OR(95% CI)	*P*-value
Continuous SPISE (per unit)	0.68 (0.59~0.78)	<0.001	0.73 (0.62~0.86)	<0.001	0.73 (0.60~0.88)	0.001
Continuous SPISE (per SD)	0.53 (0.43~0.66)	<0.001	0.60 (0.46~0.78)	<0.001	0.60 (0.44~0.82)	0.001
Q1	Ref.		Ref.		Ref.	
Q2	0.74 (0.39~1.38)	0.338	0.79 (0.38~1.64)	0.526	0.82 (0.38~1.77)	0.619
Q3	0.47 (0.26~0.87)	0.016	0.58 (0.28~1.20)	0.143	0.62 (0.28~1.39)	0.245
Q4	0.25 (0.14~0.46)	<0.001	0.33 (0.16~0.67)	0.002	0.35 (0.15~0.79)	0.011
P for trend	0.63 (0.52~0.76)	<0.001	0.69 (0.55~0.86)	0.001	0.70 (0.54~0.91)	0.008

Model 1: Unadjusted.
Model 2: Adjusted for age, sex, and LAD.
Model 3: Adjusted for age, sex, smoking, drinking, LAD, LVEF, LVEDD, LVDD, Scr, ALT, AST, PLT and RBC.
Abbreviations: AF, atrial fibrillation; ALT, alanine aminotransferase; AST, aspartate aminotransferase; CHD, coronary heart disease; CI, confidence interval; DM, diabetes mellitus; LAD, left atrial diameter; LVDD, left ventricular diastolic dysfunction; LVEDD, left ventricular end-diastolic diameter; LVEF, left ventricular ejection fraction; OR, odds ratio; PLT,

platelet count; RBC, red blood cell count; SCr, serum creatinine; SPISE, single-point insulin sensitivity estimator.

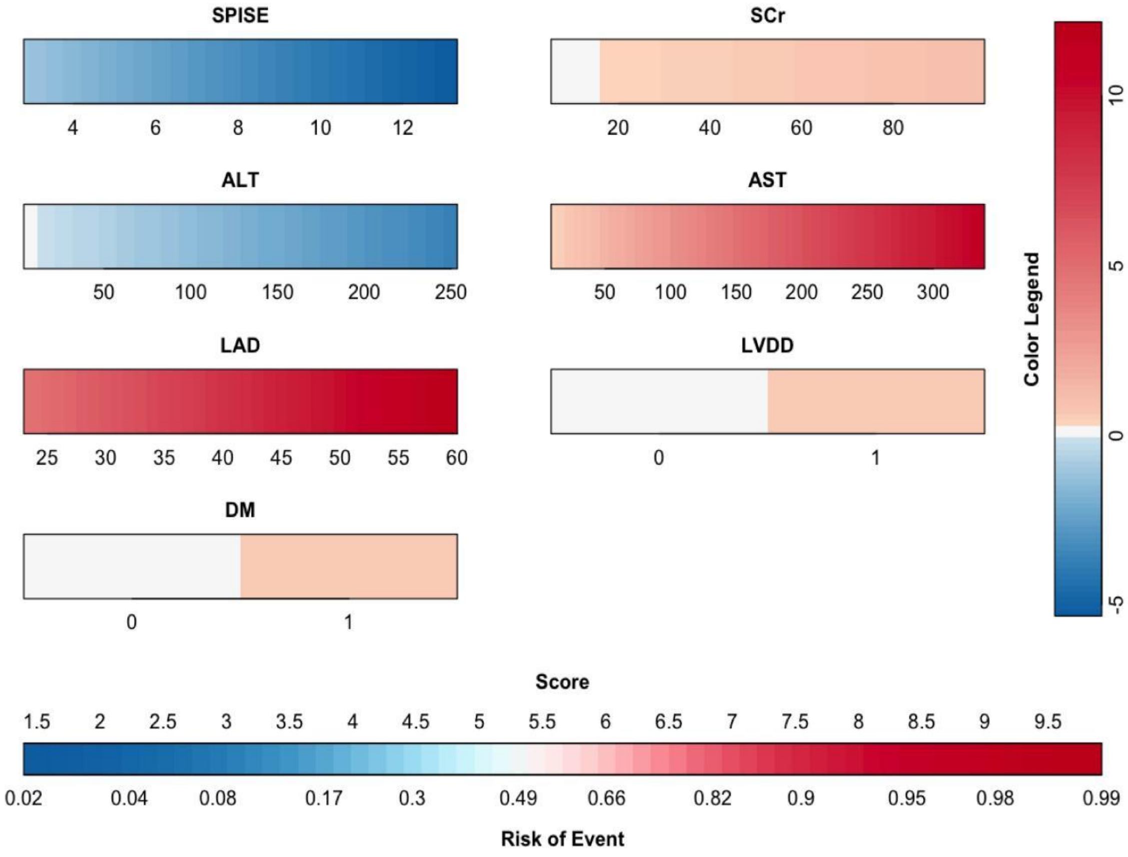

Supplementary Figure S1. Exploratory graphical representation of the fitted AF classification model. The figure is intended to visualize the fitted model and should not be interpreted as a clinically validated prediction tool.

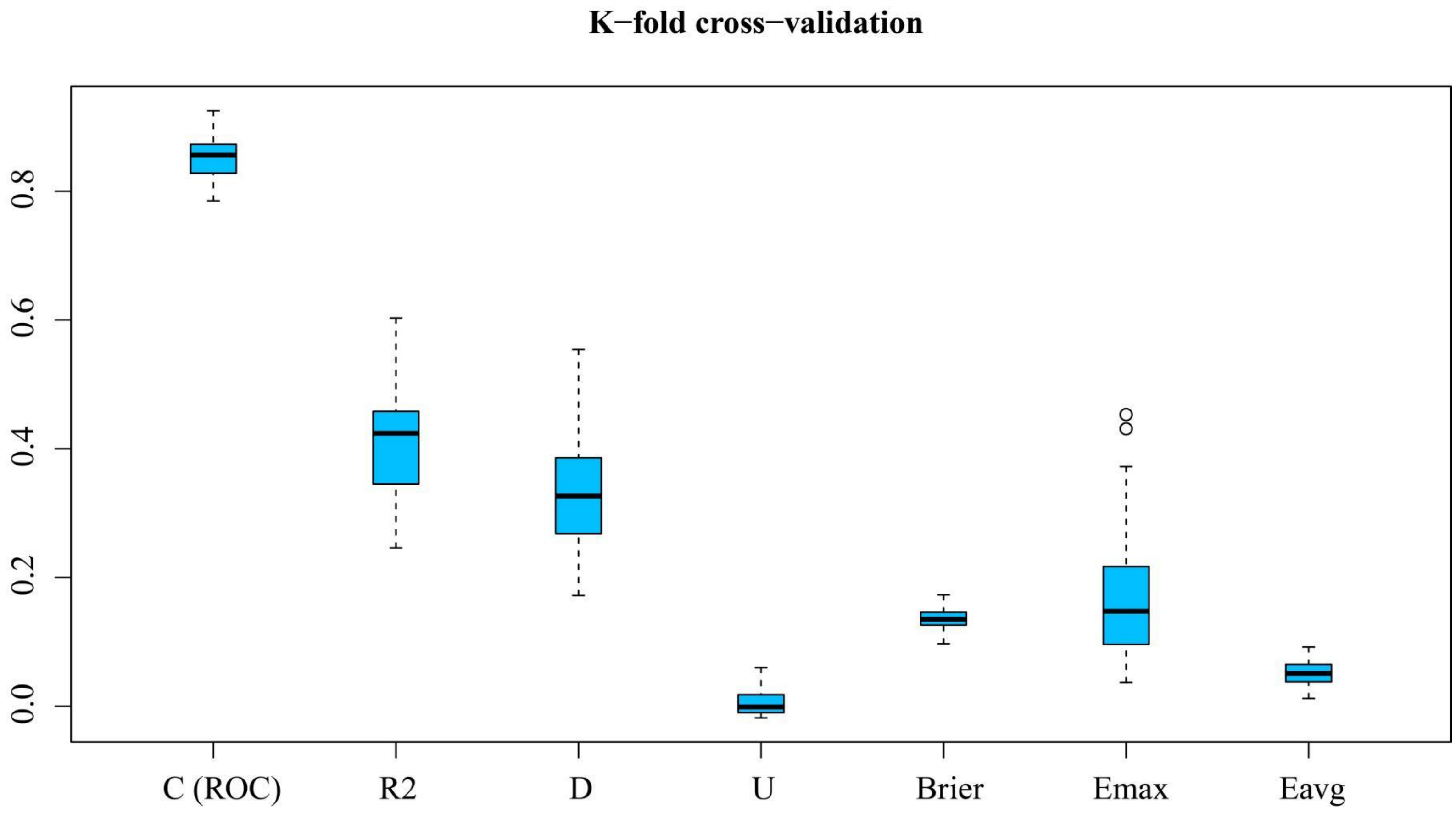

Supplementary Figure S2. Distribution of model performance metrics across repeated k-fold cross-validation.

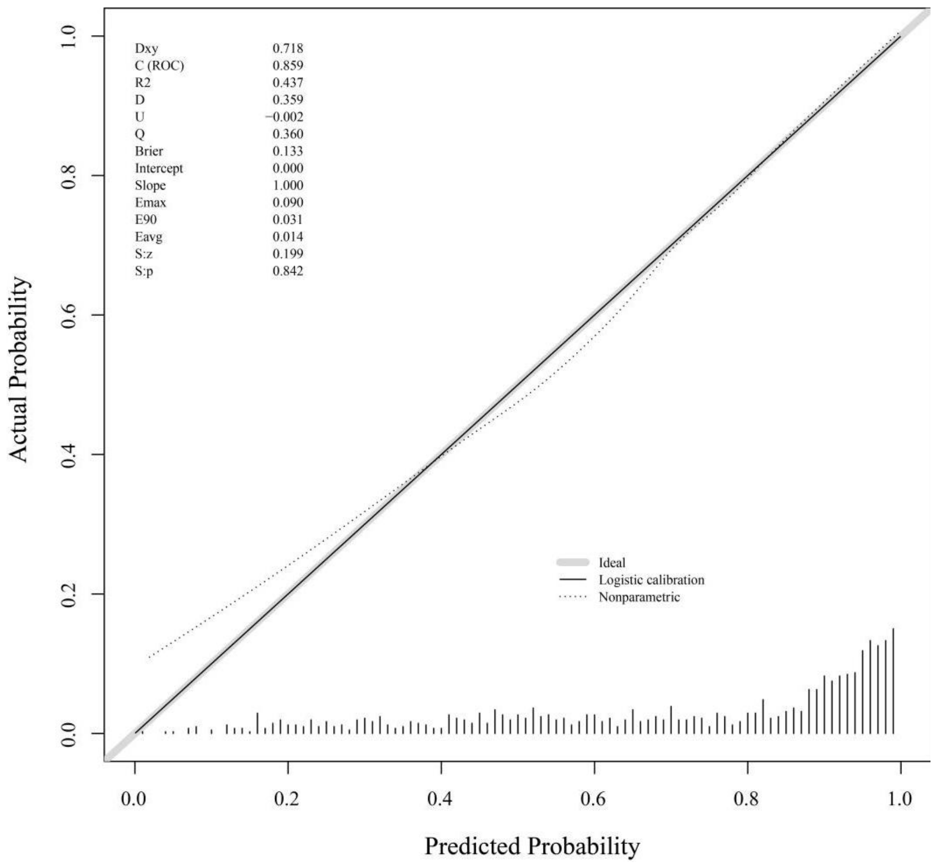

Supplementary Figure S3. Calibration curve of the final model in internal cross-validation.

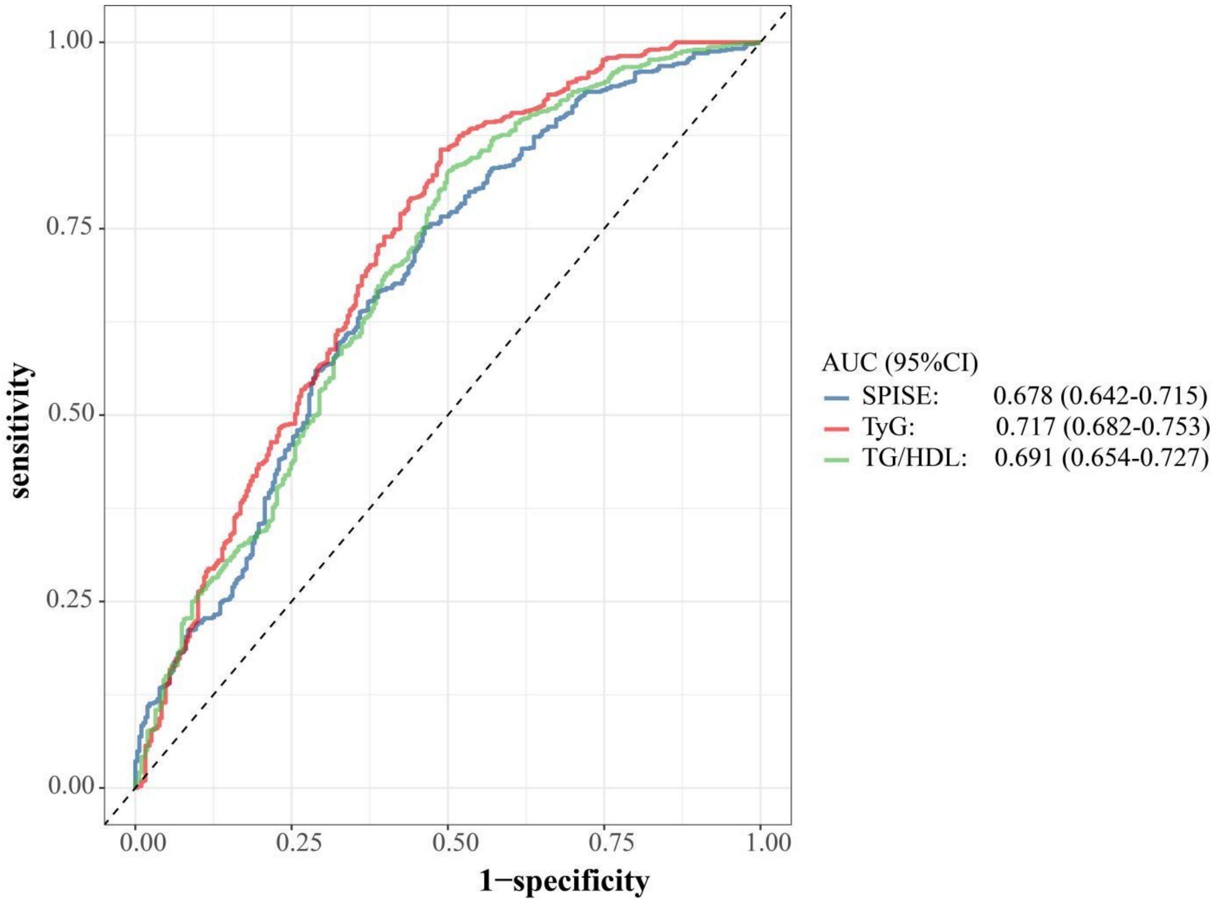

Supplementary Figure S4. Receiver operating characteristic curves comparing SPISE, TyG index, and TG/HDL-C ratio for identifying atrial fibrillation.

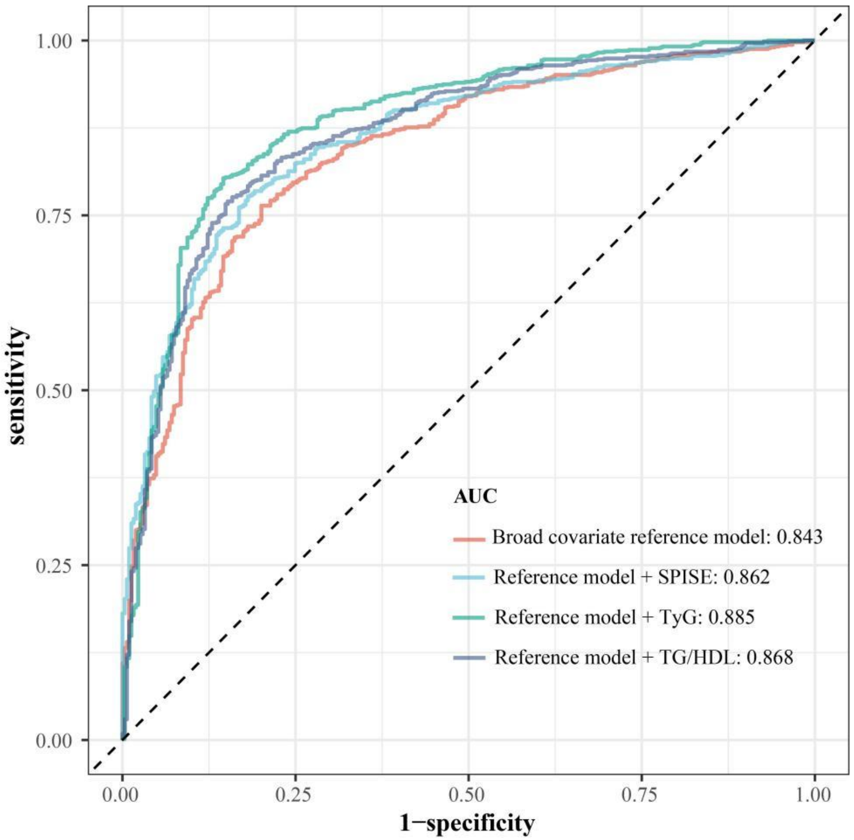

Supplementary Figure S5. Model-based ROC comparison after adding SPISE, TyG index, or TG/HDL-C ratio to the broad covariate reference model.
